# Wnt/β-Catenin Signaling Regulates Hepatitis B Virus cccDNA Levels

**DOI:** 10.3390/ijms26146942

**Published:** 2025-07-19

**Authors:** Atsuya Ishida, Sadahiro Iwabuchi, Ying-Yi Li, Kazuhisa Murai, Takayoshi Shirasaki, Kazuyuki Kuroki, Tetsuro Shimakami, Koki Nio, Kazunori Kawaguchi, Tadashi Imafuku, Satoru Ito, Taro Yamashita, Shuichi Kaneko, Hiroshi Yanagawa, Kouji Matsushima, Masao Honda, Shinichi Hashimoto

**Affiliations:** 1 Department of Clinical Laboratory Medicine, Kanazawa University Graduate School of Medical Sciences, Kanazawa 920-0942, Japan; atsuya.98617@gmail.com (A.I.); kmurai@staff.kanazawa-u.ac.jp (K.M.); takayoshi.shirasaki@gmail.com (T.S.); 2 Department of Bioinformatics and Genomics, Graduate School of Advanced Preventive Medical Sciences, Kanazawa 920-0934, Japan; iwabuchi@staff.kanazawa-u.ac.jp; 3 Department of Molecular Pathophysiology, Institute of Advanced Medicine, Wakayama Medical University, Wakayama 641-0012, Japan; imafuku@wakayama-med.ac.jp (T.I.); hashimot@wakayama-med.ac.jp (S.H.); 4 Department of Gastroenterology, Kanazawa University Graduate School of Medicine, Kanazawa 920-0934, Japan; liyingyi@staff.kanazawa-u.ac.jp (Y.-Y.L.); kkuroki@staff.kanazawa-u.ac.jp (K.K.); shimakami@m-kanazawa.jp (T.S.); nio@m-kanazawa.jp (K.N.); kawaguchi@m-kanazawa.jp (K.K.); taroy62m@staff.kanazawa-u.ac.jp (T.Y.); shuichikaneko@gmail.com (S.K.); 5 Purotech Bio Inc., Yokohama 230-0045, Japan; sr-ito@purotech-bio.com (S.I.); hyana@purotech-bio.com (H.Y.); 6 Division of Molecular Regulation of Inflammatory and Immune Disease, Research Institute for Biomedical Sciences, Tokyo University of Science, Noda 278-0022, Japan; koujim@rs.tus.ac.jp

**Keywords:** hepatitis B virus, cccDNA, DOCK11, TNKS, beta-catenin

## Abstract

Hepatitis B virus (HBV) specifically infects hepatocytes and has a complex life cycle owing to the stabilization and pooling of covalently closed circular DNA (cccDNA) in the nucleus of infected hepatocytes. We previously reported that the suppression of dedicator of cytokinesis 11 (DOCK11) decreases cccDNA and HBV-DNA levels and identified it as a new HBV therapeutic target. The DOCK11-associated gene, Wnt/β-catenin signaling regulator tankyrase (TNKS), was identified using in vitro methods; however, its function in the HBV life cycle remains unknown. Here, we used various inhibitors, antagonists, and short-hairpin RNA treatments related to TNKS signaling in HBV-infected hepatocytes. The role of TNKS-related Wnt/β-catenin signaling in the HBV life cycle was evaluated using immunoprecipitation assays with DOCK11 and bulk RNA sequencing methods. TNKS and Wnt/β-catenin signaling inhibitors significantly repressed cccDNA and HBV-DNA levels. Conversely, certain Wnt/β-catenin signaling agonists enhanced the HBV life cycle. DOCK11 directly binds to β-catenin to regulate HBV using its nuclear transport system. SKL2001, normally used as a Wnt/β-catenin signaling agonist, strongly reduced cccDNA in HBV-infected hepatocytes and in combination with entecavir predominantly eradicated HBV without cytotoxicity. Therefore, DOCK11 and other Wnt/β-catenin signaling molecules may be therapeutic targets to prevent persistent HBV infection.

## 1. Introduction

Chronic hepatitis B virus (HBV) infection is a leading cause of liver cirrhosis and hepatocellular carcinoma (HCC) worldwide. The key to persistent HBV virology is an intracellular HBV replication intermediate, called covalently closed circular (ccc) DNA, which exists as a stabilized plasmid-like molecule in the nucleus of HBV-infected cells and gives rise to progeny viruses [1]. Thus, to completely cure chronic HBV, cccDNA must be removed. A mean of around 0.1–1.0 or 10 copies per cell is detected in HBV-infected human liver chimeric mice or duck liver, respectively [2]. However, the molecular mechanisms underlying cccDNA formation and degradation, remain unclear. We previously reported that knockdown of dedicator of cytokinesis 11 (DOCK11), which encodes small GTPase regulators, strongly decreased the levels of both cccDNA and HBV-DNA levels in HBV-infected primary human hepatocyte (PXB) cells [3,4,5,6]. DOCK11 is a specific guanine nucleotide exchange factor for Rho GTPase of the cell division cycle 42 (CDC42) and cccDNA synthesis is regulated by CDC42 and activated CDC42-associated kinase 1 [5]. DOCK11 and CDC42 also regulate the retrograde trafficking of HBV from the Golgi to the endoplasmic reticulum (ER) [4]. Additionally, combination treatment with entecavir and DOCK11 depletion significantly restricts cccDNA levels compared to treatment with either drug alone in patients with chronic HBV infection [4,6]. However, the effects of DOCK11 and DOCK11-binding proteins other than CDC42 on the cccDNA life cycle remain unknown. Moreover, in the development of DOCK11-targeted inhibitors for cccDNA eradication, a detailed understanding of DOCK11-related signaling in cells will lead to the consideration of potential side effects. Here, we focused on tankyrase (TNKS) which was screened and identified as a DOCK11-associated protein using an in vitro viral method [7].

TNKS consists of two members (TNKS1 and TNKS2) and is a poly (ADP-ribose) polymerase (PARP) that regulates PAR chains via catalytic action [8]. TNKS binds to PARylate axins, which are subsequently ubiquitinated and degraded [9]. TNKS inhibitor suppresses cancer progression through inhibiting the Wnt/β-catenin signaling pathway [10]. A key feature of Wnt/β-catenin signaling is the regulated proteolysis of the downstream effector β-catenin by the β-catenin destruction complex [11]. The constituents of the β-catenin destruction complex are adenomatous polyposis coli (APC), axins, and glycogen synthase kinase 3α/β (GSK-3α/β). In the absence of Wnt pathway activation, axins binds to GSK-3β and cytosolic β-catenin at Ser45 and Ser33/37/Thr41 in which are constitutively phosphorylated by GSK-3β and targeted for degradation [12]. Axin inhibition by TNKS suppresses β-catenin phosphorylation. Conversely, the interaction of various Wnts with their receptors triggers axin detachment from the β-catenin destruction complex, leading to the inhibition of β-catenin phosphorylation [11,12]. This enhances the accumulation of nuclear β-catenin and transcription of Wnt pathway-responsive genes. Wnt/β-catenin signaling pathway modulates HBV biosynthesis [13,14]. Liver-specific β-catenin-null HBV transgenic mice lack a significant reduction in viral DNA replication and β-catenin regulates liver receptor homolog 1 (LRH1)-mediated transcription from the HBV core promoter [14]. In addition, β-catenin enhances LRH1 and farnesoid X receptor α (FXRα)-mediated activated transcription and replication of HBV in the human hepatoma Huh7 cell line [13]. However, the details regarding Wnt/β-catenin signaling and HBV replications in other HBV-infected hepatocytes remain unknown.

In the present study, we investigated whether DOCK11-associated protein TNKS and Wnt/β-catenin signaling could regulate cccDNA and HBV-DNA levels in two cell lines; HBV-infected PXB and HepG2.2.15 cells [15,16]. We also examined how various Wnt/β-catenin signaling inhibitors and agonists affect the HBV lifecycle and whether they are effective in combination with entecavir.

## 2. Results

### 2.1. Inhibition of TNKS Eliminated HBV cccDNA

**Figure 1:**
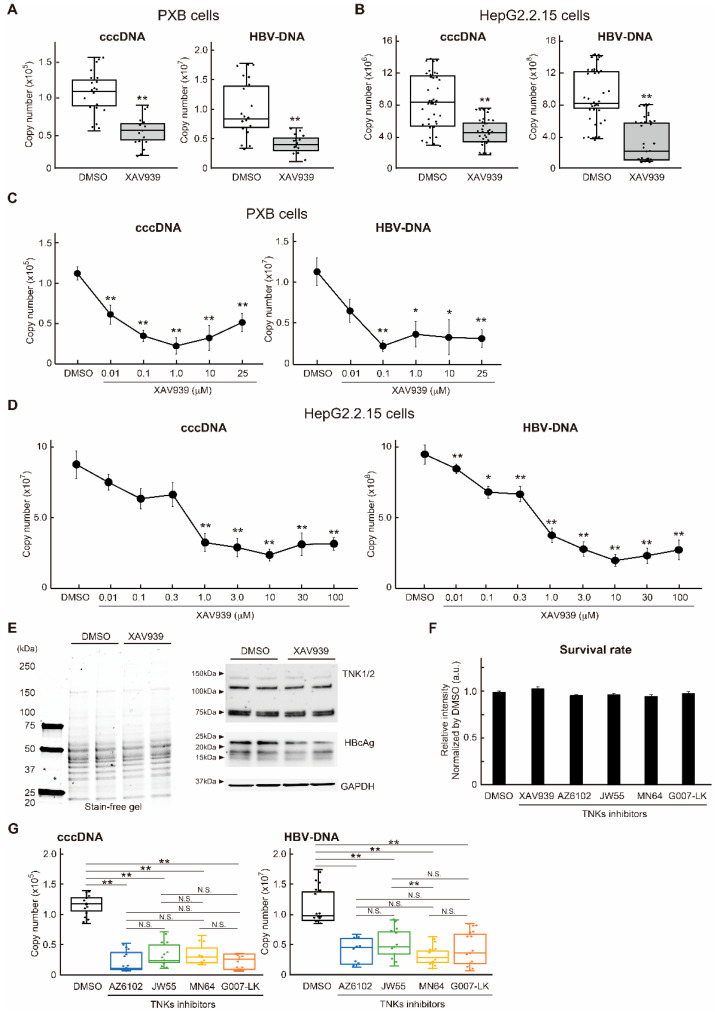
TNKS inhibitors decreased cccDNA and HBV-DNA levels. (**A**) The copy numbers of cccDNA and HBV-DNA were measured by qPCR in PXB cells. The box plot represents the median, and the box spans the interquartile range (IQR), from the 25th percentile (Q1) to the 75th percentile (Q3). Whiskers extend to 1.5 × IQR from the quartiles. To visualize the distribution of individual data points, a jitter was applied to avoid overlapping. All subsequent box-and-whisker plots follow the same convention. The concentration of XAV939 treatment was 100 nM. **, *p* < 0.01 vs. DMSO group. Number of independent cell culture batches (*N*) = 5; number of culture wells (*n*) = 16–24. (**B**) Graph shows the box-and-whisker plots of cccDNA and HBV-DNA copy numbers in HepG2.2.15 treated with DMSO or 10 μM XAV939. **, *p* < 0.01 vs. DMSO group. *N* = 8; *n* = 41–47. (**C**) Dose-dependent effects of XAV939 on cccDNA and HBV-DNA levels in PXB cells. The Y-axis indicates the raw copy number. Bars represent the mean ± S.E. *, *p* < 0.05; **, *p* < 0.01 vs. DMSO group. *N* = 3–8; *n* = 5–24. (**D**) Graph shows the dose-dependent changes in cccDNA and HBV-DNA copy numbers under DMSO or XAV939 treatment in HepG2.2.15 cells. Bars represent the mean ± S.E. *, *p* < 0.05; **, *p* < 0.01 vs. DMSO group. *N* = 5–8; *n* = 6–47. (**E**) Protein levels of TNKS, HBsAg, and GAPDH in PXB cells were determined by Western blotting. Representative blot images are shown: stain-free gel images (**left**) and corresponding immunoblot images (**right**). Each experiment was independently performed at least three times using different cell lysates. *N* = 2; *n* = 6 for each treatment group (DMSO and XAV939). (**F**) The graph shows the relative intensity (a.u.) of cell viability in each inhibitor treatment group, normalized to the DMSO-treated group. Cell viability (%) was measured by MTT assay and normalized to the mean of the DMSO group. Bars represent the mean ± S.E. No statistically significant differences were observed. *N* = 3; *n* = 8–10. (**G**) The copy numbers of cccDNA and HBV-DNA were measured by qPCR in PXB cells treated with various tankyrase inhibitors (10 nM AZ6102, 10 μM JW55, 80 nM MN64, and 50 nM G007-LK). The Y-axis indicates the raw copy number. Graph shows the box-and-whisker plots of cccDNA and HBV-DNA copy numbers in PXB cells. Notably, box-and-whisker plots are shown to illustrate the distribution of values, as sufficient variability and sample size allowed for meaningful visualization. **, *p* < 0.01 vs. DMSO or among groups as indicated. *N* = 4; *n* = 10–14. N.S.; not significant.

To investigate the potential effects of TNKS inhibitors on cccDNA and HBV-DNA, we applied 0.1 μM of XAV939 to PXB cells (Figure 1A) and 10 mM XAV939 to HepG2.2.15 cells (Figure 1B). Compared to control cells treated with dimethyl sulfoxide (DMSO), cccDNA and HBV-DNA levels were significantly lower (*p* < 0.001) in both cell lines. The DMSO concentration of culture medium in PXB and HepG2.2.15 cells was less than 0.05%. XAV939 was applied from d 1 to d 12 to HBV-infected PXB cells, and the medium was changed on d 6. Conversely, HepG2.2.15 cells were treated with TNKS inhibitors for 12 d and medium was changed on d 4 and 8. About 60% or 67% suppression of cccDNA or HBV-DNA was observed in XAV939-treated PXB cells (Figure 1A). In HepG2.2.15 cells, the cccDNA copy number of DMSO or XAV939-treatment groups was 8.5 × 10^8^ ± 6.3 × 10^6^ and 4.4 × 10^8^ ± 2.8 × 10^6^ (mean ± S.E.), respectively (Figure 1B). DNA copy number decreased by about 66% with XAV939 treatment. To examine the dose-dependent effects of XAV939 on cccDNA and HBV-DNA suppression in each cell line, we treated PXB cells with XAV939 (10 nM–25 μM) (Figure 1C) or HepG2.2.15 cells with 10 nM–100 μM (Figure 1D). XAV939 was lethal at concentrations above 25 μM in PXB cells or 100 μM in HepG2.2.15 cells, and the final DMSO concentration in each culture medium was 0.125 or 0.5%, respectively. Thus, the concentration of XAV939 that significantly reduced both cccDNA and HBV-DNA was used in subsequent experiments. The effect of XAV939 on TNK1/2 and HBsAg protein levels in PXB cells was determined using Western blotting (Figure 1E). The expression level of TNK1/2 was unaffected; however, HBcAg levels were significantly reduced. RNA-seq results showed that *TNKS* or *TNKS2* mRNA levels in HBV-infected PXB cells were slightly increased (1.08 or 1.25-fold changes, respectively) (Supplementary Data S1). To further investigate the effect of TNKS inhibition on cccDNA and HBV-DNA, we used four other representative TNKS inhibitors [17,18,19,20,21]. The appropriate concentration of each TNKS inhibitor was determined based on cell viability using the MTT assay (Figure 1F). Compared to those in the DMSO loading group, cccDNA and HBV-DNA levels were significantly decreased in the 10 nM AZ6102 [20], 10 μM JW55 [17,21], 80 nM MN64 [18] and 50 nM G007-LK [19] treatment groups (Figure 1G), strongly suggesting that TNKS is involved in regulating HBV production.

### 2.2. Identification of Differentially Expressed Genes (DEGs) Associated with TNKS Inhibition

**Figure 2:**
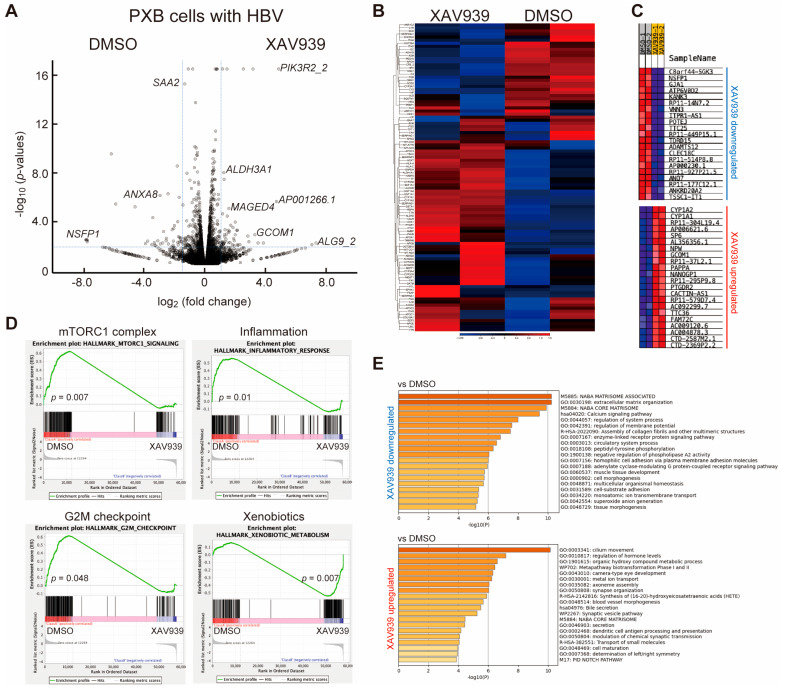
Differential expression analysis of RNA-seq datasets. (**A**) RNA-seq data were analyzed using the CLC Genomics Workbench, and the DGEs between the DMSO and XAV939 treatment groups were visualized using a volcano map. The definition of DEGs (blue dotted lines) was log_2_ FC > 1.0 and –log_10_(*p*-value) > 2.0. We used two different RNA-seq datasets obtained from different PXB cells to detect DEGs. (**B**) Heatmap analyzed using CLC Genomics Workbench. The top 100 DEGs were identified. Left two rows: XAV939 treatment group; right two rows: DMSO treatment group. The red-to-blue scale bar indicates log_2_ FC. (**C**) Heatmap plots of the top 50 upregulated or downregulated DEGs obtained using gene set enrichment analysis (GSEA). Each heatmap shows only the top 20 extracted genes. The colors are row-normalized rank-ordered gene scores such that the maximum value for each gene is plotted in red and the minimum value is plotted in blue. (**D**) GSEA revealed pathways with one representative positive enrichment and three negative enrichments after XAV939 treatment relative to DMSO controls. The genes corresponding to the red part of the heat map were highly expressed in the DMSO treatment group, and the genes corresponding to the blue part were highly expressed in the XAV993 treatment group. The signal-to-noise ratio corresponding to each gene is shown in the gray area map. (**E**) Gene Ontology (GO) analysis using Metascape showed significantly downregulated (**upper graph**) and upregulated gene functions (**lower graph**) following XAV939 treatment. Bars indicate –log_10_(*p*-value). The TPM value of the gene in XAV939 treatment group was normalized to that of the DMSO (control) group. The value > 1.5 set as threshold.

A total of 213 DEGs were detected when comparing the two XAV939 treatment groups with the two DMSO groups (Supplementary Data S2). A total of 97 upregulated and 116 downregulated genes were identified according to the criteria (−log_2_ fold change > 1.0, *p*-value < 0.05), and volcano curves were constructed to visualize the DEGs (Figure 2A). The heatmap shows the top 100 DEGs after hierarchical clustering across samples (Figure 2B), identifying the strongly upregulated and downregulated genes. This heatmap indicates that the changes in gene expression profiles in HBV-infected PXB cells treated with XAV939 were clearly different. Gene set enrichment analysis (GSEA) was performed to examine DEG profiles in detail. The advantage of GESA is that it does not use a cutoff condition for detecting DEGs and the gene sets are significantly detected using whole-gene data, even though the changes in individual genes may be relatively subtle. Figure 2C shows an excerpted heat map of the top 50 features for each culture condition for the 230,213 genes. The genes most highly upregulated by XAV939 treatment were cytochrome P450 1A2 (*CYP1A2*) and 1A1 (*CYP1A1*), which are regulated by the Wnt/β-catenin pathway [22], possibly indicating that the signaling might be related to the removal of cccDNA and HBV-DNA. Conversely, XAV939 downregulated genes, such as *NFSP1*, *GJA1*, *KANK3*, and *WNN3,* which were possible candidate genes for regulating intracellular cccDNA maintenance. However, most of the genes affected by XAV939 treatment were lncRNAs or snoRNAs. Differences in individual gene expression were negligible, and the efficiency of HBV infection in PXB cells must be considered. GSEA revealed that 41/50 gene sets were upregulated in the DMSO group, and eight gene sets were significantly enriched (nominal *p*-value < 0.05). The enrichment score (ES) line graph indicated the ES value of the gene set (Figure 2D) and the top three significantly enriched gene sets (mToRC1 complex, G2M checkpoint, and inflammatory response) were shown. In contrast, 9/50 gene sets were upregulated in the XAV939 treatment groups; however, only one gene set, xenobiotic metabolism, was significantly enriched (nominal *p*-value < 0.05). Next, we performed Gene Ontology (GO) functional annotation of the DEGs using Metascape [23]. First, we calculated the ratio of the average TPM values between the two different DMSO and XAV939 treatment groups, and over 1.5-fold higher genes under each condition were applied to Metascape. The 968 genes upregulated in the DMSO groups, that is, downregulated in the XAV939 treatment groups, were annotated, and the functions of extracellular matrix regulation, such as NABA matrisome-associated (−log_10_*p* = 10.3), extracellular matrix organization (−log_10_*p* = 10.2), and NABA core matrisome (−log_10_*p* = 9.9) were observed to be significantly enhanced. Conversely, the 816 genes upregulated in the XAV939 treatment induced the acceleration of cilium movement (−log_10_*p* = 10.1), regulation of hormone levels (−log_10_*p* = 7.1), and organic hydroxy compound metabolic processes (−log_10_*p* = 6.6) and no significant connection was observed between them. These results suggest that the XAV939 treatment of HBV-infected PXB cells might activate signals, such as cell proliferation, inflammatory response, and extracellular matrix synthesis, leading to a reduction in cccDNA and HBV-DNA.

### 2.3. Wnt/β-Catenin Signaling Regulates cccDNA and HBV-DNA Levels

**Figure 3:**
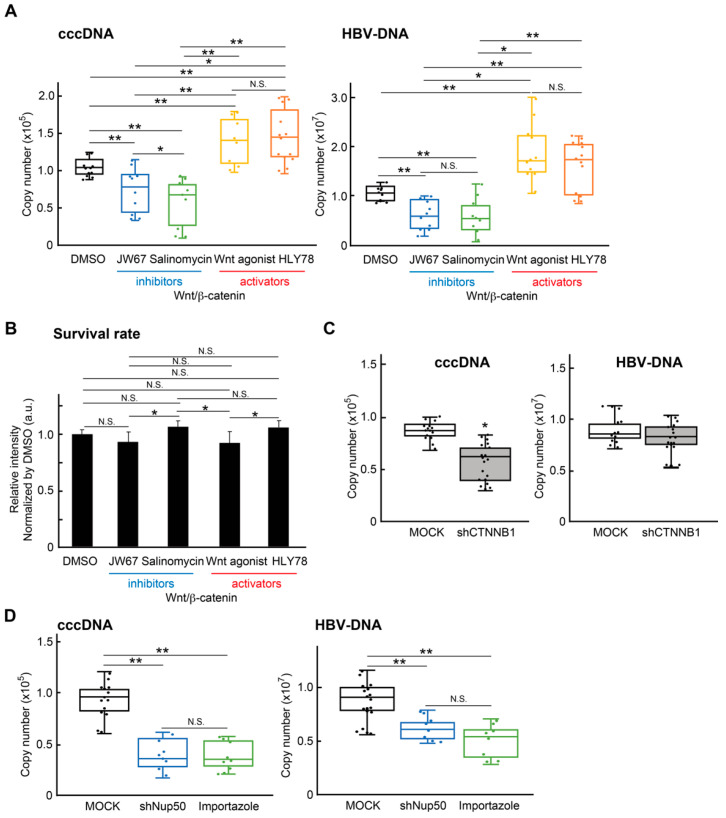
Involvement of Wnt/β-catenin signaling in HBV maintenance. (**A**) The effects of Wnt/β-catenin pathway inhibitors and activators on cccDNA and HBV-DNA were measured by qPCR in PXB cells. The Y-axis indicates the raw copy number. Box-and-whisker plots are shown to illustrate data distribution. *, *p* < 0.05; **, *p* < 0.01; N.S.; not significant. *N* = 3, *n* = 8–11. The final concentrations of each drug were as follows; 20 μM JW67, 200 nM Salinocycin, 10 μM Wnt agonist, 50 μM HLY78, according to previous reports. (**B**) The graph shows the relative cell viability (a.u.) in each inhibitor-treated group, normalized to the DMSO group. Cell viability (%) was assessed by MTT assay and normalized to the mean value of the DMSO group. Bars represent the mean ± S.E. *, *p* < 0.05; N.S.; not significant. *N* = 3, *n* = 8–11. (**C**) Graph shows the box-and-whisker plots of cccDNA and HBV-DNA copy numbers in the MOCK and shCTNNB1 group. *, *p* < 0.05 vs. MOCK group. *N* = 8, *n* = 41–47. (**D**) The copy numbers of cccDNA and HBV-DNA were measured in MOCK, shNup50 (shRNA against Nup50), and Importazole (50 μM) treatment groups. The Y-axis indicates the raw copy number. Box-and-whisker plots are shown. **, *p* < 0.01 vs. corresponding control or among groups as indicated. N.S.; not significant. *N* = 4, *n* = 4–6.

The XAV939 strongly regulates the Wnt pathway by inhibiting TNKS and subsequent destabilization of β-catenin levels [24]. Therefore, we next examined the effects of Wnt/β-catenin signaling inhibitors and activators on cccDNA and HBV-DNA levels in PXB cells. Representative inhibitors of Wnt/β-catenin signaling JW55 [25], JW67 [25], and salinomycin [26] significantly suppressed the cccDNA and HBV-DNA levels. Conversely, the activators of Wnt/β-catenin signaling, namely 10 μM Wnt agonist [27] and 50 μM HLY78 [28], strongly enhanced both levels (Figure 3A). The concentrations of each drug were not cytotoxic to the DMSO-treated groups (Figure 3B). To directly examine the relevance of β-catenin to the HBV, we applied lentiviral short hairpin RNA (shRNA) targeting β-catenin (*CTNNBL1*) to HBV-infected PXB cells. The reduction in CTNNBL1 mRNA showed a slight decrease in cccDNA, though no change in HBV-DNA (Figure 3C), suggesting that β-catenin might regulate nuclear transport of rcDNA in the early stage of HBV infection. Stabilized β-catenin possesses the ability to translocate between nucleus and cytoplasm through the nuclear pore complexes (NPCs) [29]. The β-catenin directly binds to nuclear pore complex (NPC) proteins (nucleoporins), which are similar to importin-β family members. NUP50 has been proposed to act as a cofactor for importin-α and importin-β nuclear complex import cargo [30]. Importin-β can infiltrate the HBV core dimer and empty capsids [31]. Both shRNA targeting NUP50 mRNA (shNUP50) and the inhibitor of importin-β importazole (50 μM) [32] significantly decreased of cccDNA and HBV-DNA in HBV-infected PXB cells (Figure 3D). These results suggest that Wnt/β-catenin signaling was associated with the mechanism of HBV maintenance.

**Figure 4:**
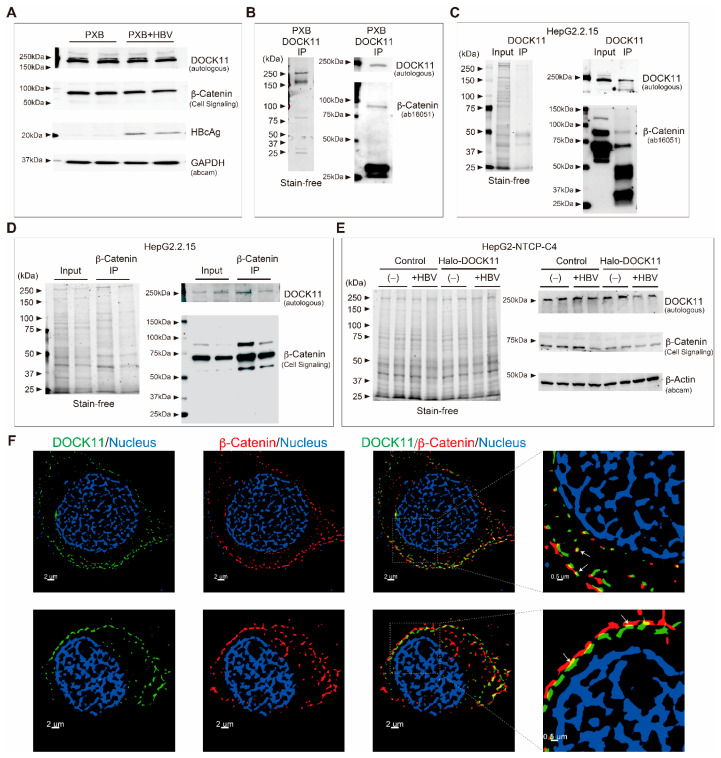
Interaction of β-catenin and DOCK11 proteins. (**A**) Protein levels of DOCK11, β-catenin, HBsAg, and GAPDH in PXB cells were determined using Western blotting. The whole blotting image obtained using stain-free gel is shown in Supplementary Figure S1. Representative blotting images for PXB control or PXB with HBV-infected groups are shown. The arrowheads indicate the size of bands (kDa). (**B**) Immunoprecipitation (IP) assays using DOCK11 in HBV-infected PXB cells. Whole gel image is shown (**left**). Western blotting using anti-DOCK11 or β-catenin antibody detected 250 kDa or 80 kDa, respectively (**right**). (**C**) IP assay using DOCK11 antibody in HepG2.2.15 cells. The whole stain-free gel image (**left**). Left lane: input; right lane: IP for DOCK11. The lysate of IP by using DOCK11 antibody was then examined for co-expression by Western blotting with β- Catenin antibodies (**right**). (**D**) IP using β-catenin antibody in HepG2.2.15 cells. Whole stain-free gel image is shown (**left**). Left lane: two inputs; right lane: two IP assays of β-catenin. DOCK11 was detected in the lysate of IP for β-catenin (**right**). (**E**) Immunoblotting of DOCK11, β-catenin, and β-actin in the cell lysate obtained from HepG2-NTCP-C4, HepG2-NTCP-C4-Halo-DOCK11 cells with or without HBV infection. Whole stain-free gel image is shown (**left**). Two representative lysates from each condition. Control; lysates from HepG2-NTCP-C4, Halo-DOCK11; lysates from HepG2-NTCP-C4-Halo-DOCK11 cells. (−); non-HBV-infected, +HBV; HBV-infected. Immunoblotting images using anti-DOCK11, β-catenin or β-Actin antibodies are shown (**right**). (**F**) Representative immunofluorescence images of HepG2.2.15 cells co-stained with DOCK11 and β-catenin antibodies. DOCK11 is shown in green, β-catenin in red, and nuclei are counterstained with DAPI (blue). Merged images demonstrate the subcellular localization and partial co-localization of DOCK11 and β-catenin. Magnified views are shown to highlight regions of co-localization. Scale bars are indicated in each image. White arrows indicate representative sites of co-localization.

We previously showed that DOCK11 is essential for the maintenance of HBV in infected PXB cells and regulates unique HBV retrograde nuclear trafficking via Golgi-to-ER transport [4,6]. To investigate whether DOCK11 was associated with Wnt/β-catenin signaling, we performed IP assays with DOCK11 and β-catenin antibody in HBV-infected PXB and HepG2.2.15 cells. The mRNA expression of *DOCK11* and *CTNNBL1* was relatively similar in control and HBV-infected PXB cells (Supplementary Data S1). As shown in Figure 4A, HBV infection did not alter DOCK11 and β-catenin protein levels. The IP assay using DOCK11 antibody in HBV-infected PXB cells and HepG2.2.15 cells clearly detected β-catenin protein (Figure 3B,C). In addition, the IP assay using β-catenin antibody also clearly detected DOCK11 protein in HepG2.2.15 cells (Figure 4D). In contrast, no correlation between DOCK11 and β-catenin expression levels was observed using HepG2-NTCP-C4-Halo-DOCK11 cell-derived lysate (Figure 4E). Immunofluorescence staining using DOCK11 and β-catenin antibody in HepG2.2.15 cells revealed co-localization of both in the cytoplasm and around the nuclear membrane (Figure 4F). Enrichment gene set analysis by using RNA-seq data indicated that no significant difference occurred for gene sets of Wnt/β-catenin signals in MOCK or shRNA for DOCK11-treated PXB cells (Supplementary Figure S2). DOCK11 regulates rcDNA transport from the cytoplasm to the nucleus, and DOCK11 and cccDNA co-localize in the nucleus [3]. Therefore, our results imply that DOCK11/β-catenin complex with rcDNA freely translocates between nucleus and cytoplasm through the NPCs.

### 2.4. Wnt/β-Catenin Agonist SKL2001 Strongly Suppressed cccDNA and HBV-DNA Levels

**Figure 5:**
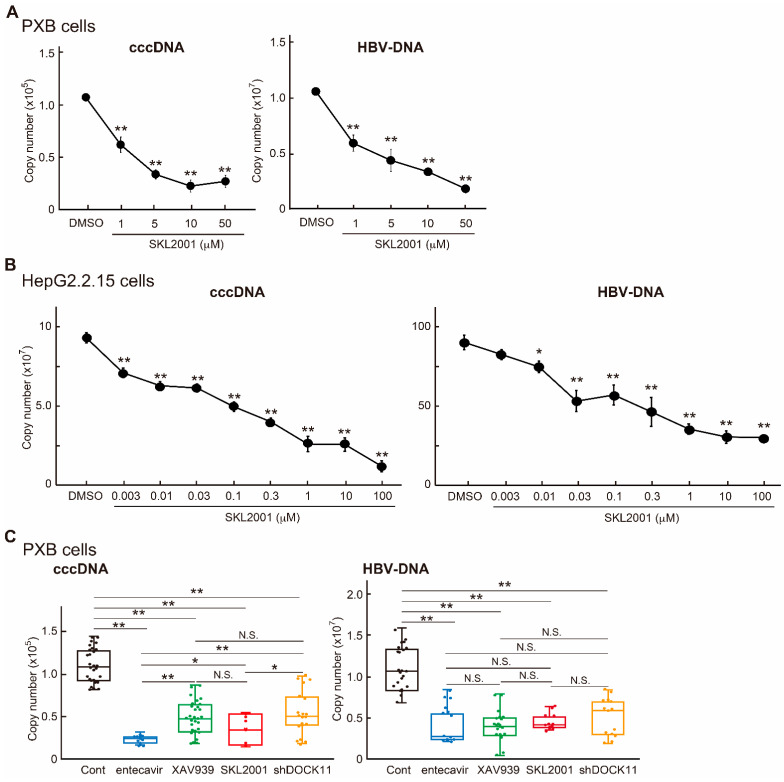
Effects of SKL2001 for eliminating cccDNA and HBV-DNA. (**A**) The effect of the Wnt/β-catenin agonist SKL2001 on cccDNA and HBV-DNA was measured by qPCR in PXB cells. The Y-axis indicates the raw copy number. Bars represent the mean ± standard error (S.E.). **, *p* < 0.01 vs. DMSO group. *N* = 3, *n* = 7–18. (**B**) Dose-dependent effects of SKL2001 on cccDNA and HBV-DNA copy numbers were assessed in HepG2.2.15 cells. The graph shows the mean ± S.E. of raw copy numbers compared to those in the 0.05% DMSO group. *, *p* < 0.05; **, *p* < 0.01 vs. DMSO group. *N* = 3, *n* = 5–8. (**C**) Graph shows the box-and-whisker plots of cccDNA and HBV-DNA copy numbers in each condition. The respective controls were purified water (for entecavir), 0.05% DMSO (for SKL2001 and XAV939), and MOCK (for shDOCK11). Comparisons were performed both against each control and among treatment groups. *, *p* < 0.05; **, *p* < 0.01; N.S., not significant. *N* = 3–4, *n* = 8–21. The final concentrations of entecavir or XAV939 were 10 nM or 100 nM, respectively.

**Figure 6:**
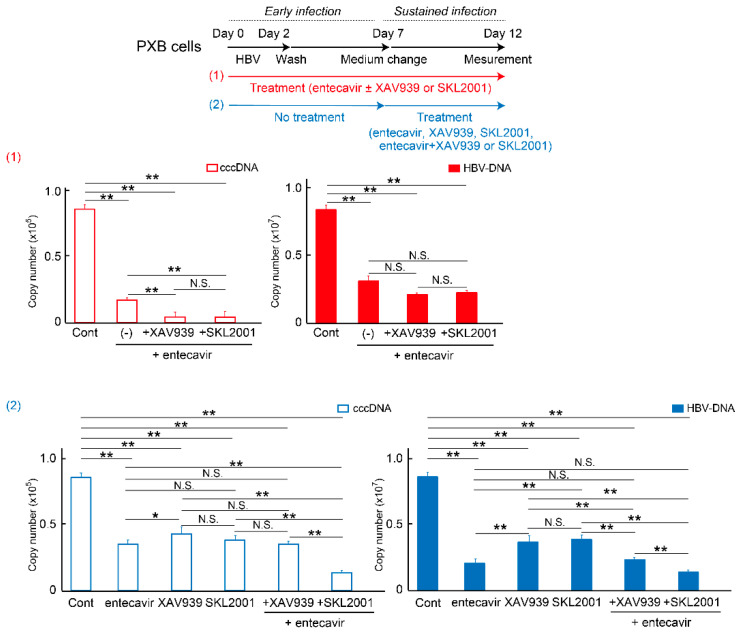
Effects of co-administration with entecavir on cccDNA and HBV-DNA levels. The upper panel illustrates the loading schedule of various inhibitors in PXB cell cultures. In the red condition (**1**), 10 nM entecavir was co-administered with either 100 nM XAV939 or 10 μM SKL2001 continuously throughout the HBV infection period. In contrast, the blue condition (**2**) involved no drug treatment during the initial 7 days of HBV infection, followed by the application of each inhibitor for up to 5 days. The lower bar graphs show the effects of these combination treatments under each culture condition. The Y-axis indicates the raw copy number. In the red group (**1**), the graph with a red border indicates cccDNA, and the red-filled graph represents HBV-DNA levels. The condition marked as (–) corresponds to treatment with entecavir alone. Bars indicate the mean ± standard error (S.E.). Asterisks (**, *p* < 0.01) indicate statistically significant differences compared to the control group. Additionally, the differences between the entecavir alone group and the combination groups with either XAV939 or SKL2001 were statistically significant. In the blue group (**2**), the blue-bordered graph shows cccDNA levels, while the blue-filled graph shows HBV-DNA levels. Bars indicate the mean ± S.E. Statistically significant differences compared to control group are denoted by *, *p* < 0.05 or ** *p* < 0.01. The comparison between the entecavir group and the entecavir plus XAV939 group showed no significant difference (N.S.), whereas a significant reduction in both cccDNA and HBV-DNA levels was observed in the entecavir plus SKL2001 group compared to the entecavir-alone group (*p* < 0.01). The number of independent culture batches was 3, and the number of wells analyzed per group ranged from 8 to 10 (*N* = 3, *n* = 8–10).

We subsequently examined the effect of another Wnt/β-catenin agonist, SKL2001 (5-[furan-2-yl]-N-[3-(1H-imidazol-1-yl)propyl]-1,2-oxazole-3-carboxamide) in HBV-infected cells. SKL2001 was originally discovered as a potent activator of the Wnt/β-catenin signaling pathway that disrupts axin/β-catenin interactions in the β-catenin destruction complex without affecting GSK3α/β kinase activity [33]. Treatment of cells with SKL2001 induces β-catenin accumulation in the cytoplasm [33,34] and it has both proliferative and inhibitory effects on cell growth [35,36]. Here, the addition of SKL2001 as a Wnt/β-catenin agonist in HBV-infected PXB and HepG2.2.15 cells resulted in a significant dose-dependent suppression of cccDNA and HBV-DNA levels, contrary to expectations (Figure 5A,B). For PXB cells, 10 μM of SKL2001 showed no cytotoxicity compared to DMSO adjusted to the same concentration (0.05% in total medium); however, 100 μM and 300 μM of SKL2001 treatment induced significant cytotoxicity in HepG2.2.15 cells (Supplementary Figure S3). Although DMSO is believed to have relatively low toxicity, the inhibition of cell proliferation induced over 100 μM SKL2001 treatment was due to high concentration of DMSO. Comparison analysis indicated that 10 nM entecavir treatment suppressed cccDNA the most, and 10 μM SKL2001 administration tended to be significantly higher (*p* = 0.015) compared to entecavir (Figure 5C). Conversely, no significant difference was observed in the HBV-DNA levels among the culture conditions. To investigate the effect of co-treatment with entecavir, each drug was administered to PXB cells from 1 d before HBV infection (d 0) to d 12 (Figure 6; red). The cccDNA but not HBV-DNA levels were significantly lower in the combination treatment groups of XAV939 or SKL2001 with entecavir compared to those in the entecavir alone treatment group. Next, we examined whether the combined effect with entecavir was also observed when each drug was added 7 d after HBV infection. The combination treatments of SKL2001 with entecavir significantly reduced cccDNA levels compared to entecavir alone; while the combination of VAX939 with entecavir had no additional effects compared to entecavir alone (Figure 6; blue). The elimination of cccDNA at the same concentration as entecavir was more efficient when it was added during the early phase of HBV infection. The combined effect of entecavir and SKL2001 treatment in reducing cccDNA and HBV-DNA levels was observed in HepG2.2.15 cells (Supplementary Figure S4).

### 2.5. DEGs of Co-Treatment Effect of Wnt/β-Catenin Agonist SKL2001 and Entecavir

**Figure 7:**
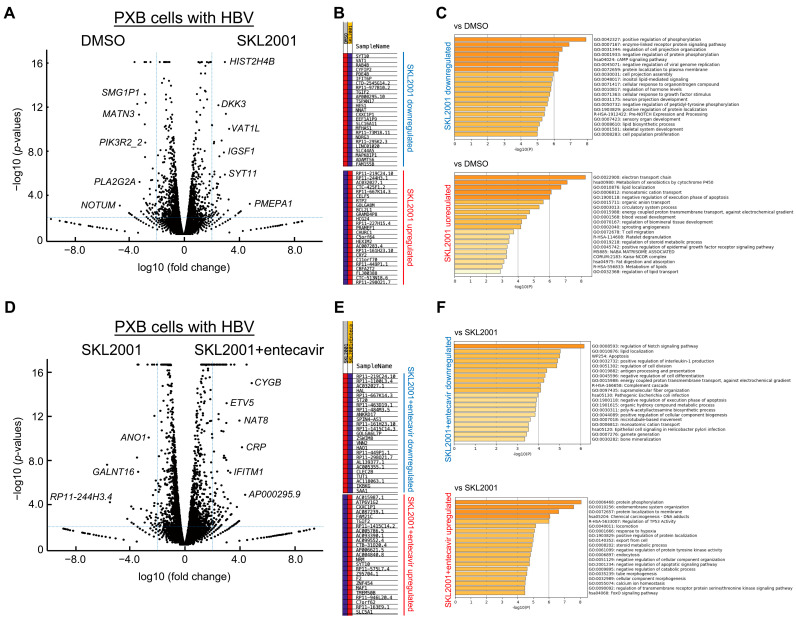
RNA-seq analysis in three different culture conditions. (**A**) RNA-seq data were analyzed by CLC Genomics Workbench, and the DEGs between DMSO and 10 μM SKL2001 treatment groups were visualized using a volcano map. The definition of DEGs (blue dotted lines) was log_2_ FC > 1.0 and −log_10_(*p*-value) > 2.0. We used two different RNA-seq datasets obtained from different PXB cells to detect DEGs. (**B**) Heatmap plots of the top 50 upregulated or downregulated DEGs obtained using gene set enrichment analysis. Each heatmap shows only the top 20 extracted genes. The colors are row-normalized rank-ordered gene scores such that the maximum value for each gene is plotted in red and the minimum value is plotted in blue. “SKL2001 downregulated” is equivalent to “DMSO upregulated,” indicating these genes might be related to the maintenance of HBV in PXB cells. (**C**) Gene Ontology (GO) analysis using Metascape showed significantly downregulated gene functions (upper graph) and upregulated gene functions (lower graph) following SKL2001 treatment. Bars indicate −log_10_(*p*-value). (**D**) Volcano map between 10 μM SKL2001 and 10 μM SKL2001 with 10 nM entecavir treatment groups. (**E**) Heatmap plots of the top 20 upregulated or downregulated DEGs obtained using GSEA enrichment analysis. “SKL2001 + Entecavir downregulated” means “SKL2001 treatment alone upregulated.” (**F**) The effect of co-treatment with SKL2001 and entecavir on signaling pathways analyzed using Metascape. Bars indicate −log_10_(*p*-value).

RNA-seq was performed on HBV-infected PXB cells to investigate the gene expression kinetics of the combined effects of SKL2001 and entecavir (Figure 7). Some studies showed that Notch is abundant in patients with HBV [37,38,39], HBx protein activates Notch signaling in HBV-HCC [40], and Notch signaling activity facilitates cccDNA transcription in HepG2.2.15.7 cells [41]. Our gene ontology analysis by using RNA-seq data indicated that 10 μM SKL2001 administration downregulated Notch signaling (R-HAS-1912422: Pre-NOTCH Expression and Processing) compared to that in the DMSO treatment group (Figure 7C). Co-treatment with SKL2001 and entecavir further suppressed Notch signaling (GO:0008593 regulation of the Notch signaling pathway) (Figure 7F), indicating that the combination of SKL2001 and entecavir may be effective in eliminating HBV. SKL2001 plus entecavir treatment enhanced protein phosphorylation (GO: 0006468), negative regulation of apoptosis (WP254, GO: 1900118, GO: 2001234), cell differentiation (GO: 0045596, negative regulation of cell differentiation), and endocytosis (GO: 0006897). The detailed involvement of each signaling pathway in cccDNA or HBV-DNA elimination is unknown; however, combination treatment did not activate any signals associated with cytotoxicity. RNA-seq results for non-HBV HepG2 and HepG2.2.15 cells treated with SKL2001 are shown in Supplementary Figure S5. SKL2001 treatment tended to enhance Wnt/β-catenin signaling; however, the effects were not significant compared to those under control culture in both cell lines. The signaling pathways accelerated by SKL2001 treatment in HepG2 and HepG2.2.15 cells were similar, indicating that the gene expression changes in HepG2.2.15 cells after the removal of HBV by SKL2001 treatment were not extremely high. Rather, SKL2001 treatment resulting in upregulated Wnt/β-catenin signaling contributed significantly to the changes in gene expression. The signaling pathways upregulated by SKL2001 treatment in HBV-infected PXB cells shown in Figure 7C differed to those in HepG2.2.15 cells, indicating a different mechanism of action for SKL2001 in HBV-infected non-cancer and cancer cells.

## 3. Discussion

The inhibition of TNKS and Wnt/β-catenin signaling reduced cccDNA and HBV-DNA levels in PXB and HepG2.2.15 cells. The tested Wnt/β-catenin agonists, except SKL2001, enhanced HBV activity. Contrary to our expectations, SKL2001 was sufficient to drastically reduce HBV infection without causing cell damage. In addition, DOCK11, which regulates the rcDNA transport from cytoplasm to nucleus [3], was co-expressed with β-catenin in HBV-infected cells, suggesting that the DOCK11/β-catenin complex with rcDNA freely translocates between nucleus and cytoplasm through the NPCs. The TNKS inhibitor, XAV939 and Wnt agonist, SKL2001 both reduced cccDNA in PXB cells to less than 25% of the controls at the maximum levels (Figure 1A,C). Therefore, reduced cccDNA might not be reflected by the cell division but reflected by the reduced copy number in cells. PXB cells could be allowed to divide once after being isolated from the liver.

The association of β-catenin with the HBV lifecycle in normal hepatocytes may be different from that in cancer cells. HepG2 cells express a stabilized mutant β-catenin and the excessive activation of Wnt/β-catenin signaling is reported [42,43]. Wnt/β-catenin signaling is activated in normal hepatocytes, though it is aberrantly activated in HCC [44,45,46,47]. Approximately 8–30% of somatic mutations of β-catenin protein in HCC prevent its degradation, leading to the accumulation of mutated β-catenin in cytosol and transfer to the nucleus to upregulate Wnt target genes [45,47]. β-catenin mutations are also detected in HBV-HCC [44,48] and poor survival is observed in patients with HCC with mutated β-catenin [44]. Cellular micro RNAs (miRNAs) regulate the expression and replication of HBV genes [46] and mutated HBx modulates Wnt/β-catenin signaling by affecting regulatory non-coding RNAs (ncRNAs) including miRNAs and ling ncRNAs [49,50]. However, HBx deletion does not affect the ability of HBV to increase in β-catenin signaling in HepG2 cells [43]. Overexpression of wildtype β-catenin in the human hepatoma cell line Huh7 slightly increased HBV-DNA levels; however, the effects on cccDNA levels were not evaluated [13]. So far, the effects of wild-type β-catenin or mutant β-catenin on cccDNA levels in cancer cells and other tumor tissue cells is unclear. Therefore, a detailed mechanism underlying β-catenin-mediated regulation of the HBV lifecycle remains unknown. In this study, the effect of Wnt/β-catenin signaling inhibitors on cccDNA levels in HBV-infected PXB cells and the HBV-expressing cancer cell line HepG2.2.15 cells were identical in vitro studies. RNA-seq data showed XAV939, a Wnt inhibitor, altered gene expression related to extracellular matrix and inflammatory responses, which may disrupt the cellular environment supporting HBV persistence.

Although the analysis did not reach statistical significance, the enrichment score showed a decreasing trend upon XAV939 treatment, consistent with its known inhibitory effect on the Wnt/β-catenin pathway. HBV infection profoundly affects multiple cellular pathways, notably causing marked downregulation of cell cycle and inflammatory response-related signaling. These dominant changes likely overshadow the differences in Wnt/β-catenin signaling, potentially reducing the apparent significance of the pathway in our GSEA results.

On the other hand, SKL2001, a Wnt activator, also reduced HBV levels. The Wnt/β-catenin agonist SKL2001, or 5-(Furan-2-yl)-N-(3-(1H-imidazol-1-yl)propyl)-1,2-oxazole-3-carboxamide, was first identified from a screening of 270,000 compounds as a small compound that disrupts the β-catenin destruction complex [33]. This inhibition of β-catenin phosphorylation at Ser33/37/Thr41/Ser45 may be involved in the SKL2001-mediated β-catenin stabilization without affecting the phosphorylation of GSK-3β at Ser9. Thus, SKL2001 would exert more specific Wnt modulating activity without affecting multiple other GSK3β-mediated signaling pathways.

The underlying mechanisms of these contradict results had not been resolved so far; however, preliminary results showed that the different modes of action of XAV939 and SKL2001 on the binding DOCK11 and β-catenin (Supplementary Figure S6B). SKL2001 inhibited the binding of DOCK11 and β-catenin, whereas XAV939 did not. Interestingly, HBV infection increased the binding of DOCK11 and β-catenin, and HBV capsid protein formed the complex with DOCK 11 that we previously reported (Supplementary Figure S6A) [4]. Although SKL2001 stabilizes β-catenin, it inhibits the binding of β-catenin to DOCK11. DOCK11 formed complex with HBV capsids that include rcDNA. Therefore, SKL2001 might suppress HBV infection.

In addition, RNA-seq data showed that SKL2001 inhibited Notch signaling. Wnt and Notch are both essential signaling for organ development. Both two signals cross-talked to each other, and it is reported that the upregulation of Wnt signaling suppressed Notch pathway [51]. Notch is abundantly expressed in patients with HBV [39], and involved in cccDNA transcription [38]. The expression patterns of Notch signaling were reduced in the presence of entecavir in HepG2.2.15.7 cells [37]. Therefore, the suppression of Notch signaling by SKL2001 might partially explain the reduced HBV by SKL2001 treatment.

Collectively, these results indicate that both inhibition and activation of Wnt signaling can impair HBV maintenance via distinct downstream mechanisms. Further detailed studies should be warranted of the effects of XAV939 and SKL2001 in normal, HBV-infected, or cancer-derived hepatocytes.

## 4. Materials and Methods

### 4.1. Cell Culture

The human liver cancer HepG2.2.15 cell line was authenticated by DNA fingerprinting in 2016. The cells were cultured onto the Collagen I coated culture well (AGC TECHNO GLASS Co. Ltd., Shizuoka, Japan) and maintained in Dulbecco modified Eagle medium/F-12, GlutaMax supplement (Thermo Fisher Scientific, Waltham, MA, USA) with 10% fetal bovine serum (BSA; Hyclone Laboratories LLC, Logan, UT, USA), 100 U/mL penicillin and 100 μg/mL streptomycin (Thermo Fisher) and 400 μg/mL G418, (Geneticin; Thermo Fisher). Primary human hepatocytes (PXB cells), HBV, and culture medium were purchased from PhoenixBio Co. Ltd. (Hiroshima, Japan). The transported cell lines were incubated for 6 h and then infected with HBV at 5 GEq/cell in the presence of 5% polyethylene glycol 8000 (Hampton Research, Aliso Viejo, CA, USA) for 1–2 d at 37 °C in a humidified incubator in an atmosphere of 5% CO_2_. After removing the supernatant, the cells were maintained in culture medium with or without inhibitors or agonists. Four–five days after the first medium change, the supernatant was replaced with fresh culture medium, with or without each drug. Cells were used for subsequent experiments, 10–12 d after HBV infection.

### 4.2. Quantification of cccDNA and HBV-DNA

Total DNA was extracted from the cells using SMITEST EX-R&D (Medical & Biological Laboratories Co. Ltd., Tokyo, Japan) according to the manufacturer’s protocol. The concentration of the extracted DNA was measured using a NanoDrop spectrophotometer (Thermo Fisher). The cccDNA was generated from 1200 ng of DNA using Plasmid-Safe ATP-dependent DNase (Lucigen, Middleton, MD, USA) [52]. The 15 μL of 1200 ng DNA, 2 μL of 10 × Plasmid-safe buffer, 2 μL of 25 mM ATP solution, 30 U (3 μL) of Plasmid-Safe ATP-dependent DNase were incubated at 37 °C for 45 min, followed by 70 °C for 30 min. After cooling at 4 °C, 8 μL of cccDNA solution or same amount of DNA, 10 μL of TaqMan Fast Universal PCR Master Mix (Applied Biosystems, Waltham, MA, USA), 0.5 μL of 25 μM HBV forward primer (5′-ACTCACCAACCTCCTGTCCT-3′), 0.5 μL of 25 μM HBV reverse primer (5′-GACAAACGGGCAACATACCT-3′) and 1 μL of 25 μM FAM/ZEN/IBFQ (5′-/56-FAM/TATCGCTGG/ZEN/ATGTGTCTGCGGCGT/3IABkFQ/-3′, purchased from Integrated DNA Technologies Inc, Coralville, IA, USA) were mixed well. After 120 s at 50 °C and 140 s at 95 °C, 50 cycles (95 °C for 3 s and 60 °C for 30 s) of polymerase chain reaction (PCR) amplification were performed for HBV detection. From the standard HBV curve, we calculated the raw HBV copy number and normalized it for each culture condition.

### 4.3. Western Blotting

Total protein was extracted from cells and boiled at 95 °C for 5 min in a loading buffer of Laemmli sample buffer with β-mercaptoethanol (Bio-Rad Laboratories Inc, Hercules, CA, USA). Cell lysates of HepG2-NTCP-C4 and Dox-inducible HepG2-NTCP-C4-Halo-DOCK11 cells with or without HBV infection were obtained from Prof. M. H. at Kanazawa University [4]. Each sample was loaded onto Mini-PROTEAN TGX stain-free gel (4–20% or 7.5%, Bio-Rad) with running buffer (Tris/glycine/SDS buffer, Bio-Rad). Electrophoresis was performed at 200 V for 30 min and the stain-free gel was photographed using a protein gel imaging system (ChemiDoc-Touch Imaging system, Bio-Rad) for detection of total proteins. The gel was transferred using Trans-Blot Turbo Transfer Pack (Bio-Rad) at 2.5 A for 7 min and the transferred membrane was cut into the targeted molecule sizes. After blocking with EveryBlot Blocking buffer (Bio-Rad) for 15 min, the membrane was incubated with the primary antibody overnight at 4 °C, followed by three washes with Tris-Buffered Saline and 0.05% Tween-20 (TTBS) buffer. The primary antibodies were anti-TNKS (ab227471, 1:500, Abcam plc, Cambridge, UK), anti-hepatitis B virus core antigen (3HB17, 1:100, HyTest Ltd., Turku, Finland), anti-GAPDH (ab9483, 1:1000, Abcam), anti-DOCK11 (autologous, 100 μg, obtained from Prof. K.M. in Tokyo University of Science), anti-DOCK11 (GTX55982, 1:1000, GeneTex Inc, Irvine, CA, USA), anti-β-catenin (#9582, 1:1000, Cell Signaling Technology Inc, Danvers, MA, USA), anti-β-catenin (ab16051, 1:1,000, Abcam) and anti-β-actin (ab8226, 1:1000, Abcam). After three washes, the secondary goat anti-rabbit or anti-mouse IgG antibodies (170-6515 or 170-6516, 1:2000, Bio-Rad) were applied at room temperature (RT) for 1 h. After washing with TTBS, an appropriate amount of Clarity Western ECL Substrate (Bio-Rad) was added to the membrane, and the membrane was placed on a ChemiDoc Touch Imaging system (Bio-Rad) to capture chemiluminescence in auto-exposure mode.

### 4.4. Cell Proliferation Assay

The Cell Proliferation Kit I (MTT based, Roche, Basel, Switzerland) was used for measurement of cell proliferation with following the manuals. In brief, 10 or 20 μL of the MTT labeling reagent was added to each well after cells were cultured in 48 or 24–well cell plate. The culture plate was incubated for 4–5 h at 37 °C, and then 100 μL of the solubilization buffer was added. The absorbance of purple formazan crystals in each well was measured using a GloMax Discover Microplate Reader (Promega, Madison, WI, USA). The survival rate (arbitrary unit, a.u.) in each culture condition was calculated by normalization to the intensity obtained by the wavelength >650 nm in the DMSO-treated culture (control).

### 4.5. RNA Sequencing (RNA-seq)

Total RNA in cultured cells was obtained using the RNeasy Mini Kit (Qiagen, Hilden, Germany) according to the manufacturer’s instructions. RNA quality was evaluated using an Agilent 4200 TapeStation (Agilent Technologies, Santa Clara, CA, USA), and RNA concentration was measured using a Qubit 2.0 Fluorometer (Thermo Fisher). A total of 1000–4000 ng of RNA from each sample was used, and libraries for sequencing were constructed using the TruSeq Stranded mRNA Kit (Illumina, Inc., San Diego, CA, USA) according to the manufacturer’s protocol. The concentration of the libraries was estimated using a KAPA Library Quantification Kit (Roche). The average library size was 350 bp. High-throughput sequencing of the samples was performed using the NextSeq 500/550 High Output Kit v2.5 (Illumina, 75 cycles pair-end, 40/40 cycles). The average number of sequence reads per sample was 23,333,374 in PXB cells and 20,649,133 in HepG2.2.15 cells. Bulk RNA-Seq results were analyzed using the CLC Genomics Workbench Version 12.0.2 (Filgen Inc., Nagoya, Japan). The raw transcript per million (TPM) data obtained from RNA-seq are provided in Supplementary Materials. Target gene sets were analyzed using gene set enrichment analysis (GSEA) [53] and the gene ontology enrichment analysis tool Metascape v3.5 [23].

### 4.6. Short-Hairpin RNA (shRNA)

The MISSION LentiPlex Human shRNA pooled library (Sigma-Aldrich Co., St. Louis, MO, USA) was used to generate lentiviruses with shRNA plasmids targeting CTNNBL1 and NUP50 in PXB cells. The validated clone ID of each particle were TCRN0000290262 (0.74 × 10^7^ TU/mL) and TRCN0000160160 (1.5 × 10^7^ TU/mL). As a negative or positive control, MISSION TRC2 Control Transduction Particle puro Non-Mammalian shRNA#1 (SHC202V, 4.6 × 10^7^ TU/mL) or MISSION TRC2 Control Transduction Particle puro-CMV-TurboGFP (SHC203V, 1.6 × 10^8^ TU/mL) was used. PXB cells were infected with hexadimethrine bromide (8 μg/mL) and four multiplicity of infection (MOI) of each particle for 1 d. Virus-containing media were aspirated and fresh complete media were added, and the cultures were incubated for 10 d with medium change at d 5. Total RNA was extracted using the RNeasy Mini Kit and purified RNA samples were processed into single-stranded complementary DNA (cDNA) using oligo (dT) and random primers (Takara Bio, Shiga, Japan) and SuperScript III Reverse Transcriptase (Invitrogen, Grand Island, NY, USA). The DNA concentration was measured using a NanoDrop and Qubit 2.0 Fluorometer. Synthesized cDNA was amplified by real-time PCR system QuantStudio3 (Thermo Fisher) using SYBR Green (Takara Bio) with specific primers; human CTNNBL1-forward (3′-ATGGACGTGGGCGAACTTC-5′) and human CTNNBL1-reverse (5′-TTTGTTTCCGACGCATCTTCT-3′) or human NUP50-forward (5′-TCTGGAGGAGGACGCTTTTCT-3′) and human NUP50-reverse (5′-GGGGCACTGGTTATGTTGTTT-3′) or human GAPDH-forward (5′-GCGCCCAATACGACCAA-3′) or human GAPDH-reverse (5′-CTCTCTGCTCCTCCTGTTC-3′) to confirm the validated shRNA knockdown efficiency.

### 4.7. Immunoprecipitation (IP) Assay

The IP assays of DOCK11 or β-catenin were performed using the Universal Magnetic CO-IP kit (Active Motif Inc, Carlsbad, CA, USA), following the manufacturer’s instruction. Briefly, cultured cells were harvested as cell pellets, and whole-cell extraction was performed. Cell lysates were incubated with 10 μg of anti-rabbit DOCK11 antibody (A301-638A, Bethyl Laboratories Inc, Montgomery, TX, USA) or 10 μg of anti-β-catenin (#9582, Cell Signaling Technology) or rabbit IgG overnight at 4 °C on a rotator. After centrifuging, the liquid was collected and Protein G magnetic beads were added, and then it was incubated for 3 h at 4 °C on a rotator. After subsequent washing four times with complete/CO-IP buffer (Active Motif), bead pellets were resuspended in the loading buffer containing β-mercaptoethanol (Bio-Rad) and boiled at 95 °C for 5 min before Western blotting. The cell lysate obtained by IP with rabbit IgG was used as the control (input).

HepG2.2.15 cells were seeded in 10 cm dishes at a density of 5 × 10^5^ cells per dish. After 16 h, cells were transfected with 8 µg of Halo-DOCK11 plasmid using Lipofectamine 3000 (Invitrogen). Twenty-four hours post-transfection, cells were treated with either 0.1% DMSO (vehicle), SKL2001 (10 µM), or XAV939 (10 µM) for an additional 48 h. Total protein was extracted using IP lysis/wash buffer (25 mM Tris, 150 mM NaCl, 1 mM EDTA, 1% NP-40, 5% glycerol; pH 7.4; Thermo #26149). In some experiments, HepG2-NTCP-C4 cells were similarly seeded and transfected with Halo-DOCK11 plasmid. After 24 h, cells were infected with HBV virions (6 × 10^6^ genome equivalents per cell) derived from HepAD38 cells in the presence of 4% PEG8000 and 2% DMSO for 16 h. Cells were then washed three times with PBS and cultured in DMEM/F-12 medium supplemented with GlutaMAX (Thermo Fisher Scientific) for an additional 36 h. Protein extraction was performed as described above.

For IP, cell lysates were incubated with Halo-Trap magnetic particles M270 at 4 °C for 1 h followed by incubation with Protein G magnetic beads. Beads were washed three times with IP lysis/wash buffer, resuspended in 30 µL SDS sample buffer, and boiled at 95 °C for 5 min to elute immunocomplexes. The supernatants were analyzed by immunoblotting. Expression of Halo-DOCK11, β-catenin, and HBc was assessed by immunoblotting using mouse anti-Halo (Promega), rabbit anti-β-catenin (#9582, Cell Signaling Technology), and rabbit anti-HBc (BCL-ABPC-01, Beacle, Inc., Kyoto, Japan) antibodies, respectively.

### 4.8. Immunofluorescence Microscopy and Fluorescence Imaging

HepG2.2.15 cells were cultured onto the Collagen I coated cover glasses (AGC TECHNO GLASS Co.) in culture-wells, and cover glasses were rinsed once using PBS with 10% BSA (washing buffer) and fixed for 30 min with 4% polyformaldehyde (PFA; FUJIFILM Wako Pure Chemical Co., Osaka, Japan). The cover glasses were then permeabilized using 1% Triton 100-X (Sigma-Aldrich) in PBS at RT for 10 min. The cover glasses were blocked with a blocking buffer Blocking One-P (NACALAI TESQUE Inc, Kyoto, Japan) for 30 min at 37 °C. The primary antibodies anti-rabbit polyclonal DOCK11 (1:200, autologous or 1:200, GeneTex), were applied overnight at 4 °C. The secondary antibodies, anti-rabbit Alexa Fluor 488 (1:2000, Thermo Fisher) were incubated for 30 min at 37 °C. Then, the same procedures were applied to the DOCK11-stained cover glasses using primary antibodies anti-mouse monoclonal β-Catenin (ab237983, 1:100, Abcam) and the secondary antibody anti-mouse Alexa Fluor 594 (1:2000, Thermo Fisher) under shading conditions. The SlowFade Diamond Antifade Mountant with DAPI (Thermo Fisher) and an encapsulating agent were added to the cover glasses, and images were acquired using ZEISS LSM900 Airyscan 2 (Carl Zeiss, Oberkochen, Germany). The acquisition conditions were 63 × oil lens, excitation channels; 405, 488, 561, emission channels; DAPI, Alexa Fluor 488 and Alexa Fluor 594, pinhole to 1 Airy Unit, master gain; 600–800, digital offset; 0, digital gain; 3.0–7.0, averaging 4×, scan speed; Max, z-stack mode; interval 1 μm and 17 slices.

### 4.9. Statistical Analyses 

Data were collected in Microsoft Excel and statistically analyzed using the data analysis tool. Results are presented either as box-and-whisker plots or as mean ± standard error (S.E.), depending on the type of analysis. For comparisons between two groups, an unpaired Student’s *t*-test was applied. For multiple comparisons, one-way analysis of variance (ANOVA) followed by Tukey’s Honestly Significant Difference (HSD) post hoc test was used. Statistical significance was defined as *p* < 0.05.

## 5. Conclusions

Treatment with XAV939 or SKL2001 inhibited of Wnt/β-catenin signaling via reducing cccDNA and HBV-DNA levels in HBV-infected PXB and HepG2.2.15 cells. The combined effects of these agents with entecavir are promising. Thus, XAV939 and SKL2001 may be novel drug candidates for eliminating HBV by suppressing HBV-derived HCC progression.

## Data Availability

All raw data included in this article are accessible to competent researchers upon adequate request.

## Supplementary Materials

The following supporting information can be downloaded at https://www.mdpi.com/article/10.3390/ijms26146942/s1.

## Abbreviations

cccDNA, covalently closed circular DNA; DOCK11, Dedicator of Cytokinesis 11; HBV, Hepatitis B virus; TNKS, Tankyrase
