# Correction: Ishida et al. Wnt/β-Catenin Signaling Regulates Hepatitis B Virus cccDNA Levels. *Int. J. Mol. Sci.* 2025, *26*, 6942

**DOI:** 10.3390/ijms27114908

**Published:** 2026-05-29

**Authors:** Atsuya Ishida, Sadahiro Iwabuchi, Ying-Yi Li, Kazuhisa Murai, Takayoshi Shirasaki, Kazuyuki Kuroki, Tetsuro Shimakami, Kouki Nio, Kazunori Kawaguchi, Tadashi Imafuku, Satoru Ito, Taro Yamashita, Shuichi Kaneko, Hiroshi Yanagawa, Kouji Matsushima, Masao Honda, Shinichi Hashimoto

**Affiliations:** 1 Department of Clinical Laboratory Medicine, Kanazawa University Graduate School of Medical Sciences, Kanazawa 920-0942, Japan; 2 Department of Bioinformatics and Genomics, Graduate School of Advanced Preventive Medical Sciences, Kanazawa 920-8640, Japan; iwabuchi@staff.kanazawa-u.ac.jp; 3 Department of Molecular Pathophysiology, Institute of Advanced Medicine, Wakayama Medical University, Wakayama 641-8509, Japan; 4 Department of Gastroenterology, Kanazawa University Graduate School of Medicine, Kanazawa 920-8640, Japan; 5 Purotech Bio Inc., Yokohama 230-0027, Japan; 6 Division of Molecular Regulation of Inflammatory and Immune Disease, Research Institute for Biomedical Sciences, Tokyo University of Science, Noda 278-8510, Japan; koujim@rs.tus.ac.jp

In the original publication [1], there were errors in Figures 1, 4, 5, 7, S3 and S6. The corrected figures and legends appear below.

In Figure 1 (specifically panels 1B and 1D), the cell line name was incorrectly labeled as “HepG2.15.2” and has been corrected to “HepG2.2.15.” In addition, axis lines were added to the x-axis to improve readability.

In Figure 4, panel F was incorrectly uploaded and has now been replaced with the correct panel. Furthermore, the cell line name in panels 4C and 4D has been corrected from “HepG2.15.2” to “HepG2.2.15.” Additionally, the legend for Figure 4F was revised as the panel was incorrectly described as an “Ortho view of Z-stack images,” and the scale bars and arrow colors were inaccurately stated.

In Figure 5B, the cell line name has been corrected from “HepG2.15.2” to “HepG2.2.15.” Axis lines were also added to the x-axis to improve readability.

In Figure 7C, an incorrect Metascape panel was included and has now been replaced with the correct version.

In Supplementary Figure S3, axis lines were added to the x-axis to improve readability. In Supplementary Figure S6, the cell line name has been corrected from “HepG2.15.2” to “HepG2.2.15,” and axis lines were added to the x-axis. Consequently, the Supplementary Figures file has been replaced with an updated version (Supplementary_Figures-mod.pdf), in which the corrected figures have been incorporated.

The authors state that the scientific conclusions are unaffected. This correction was approved by the Academic Editor. The original publication has also been updated.

**Figure 1:**
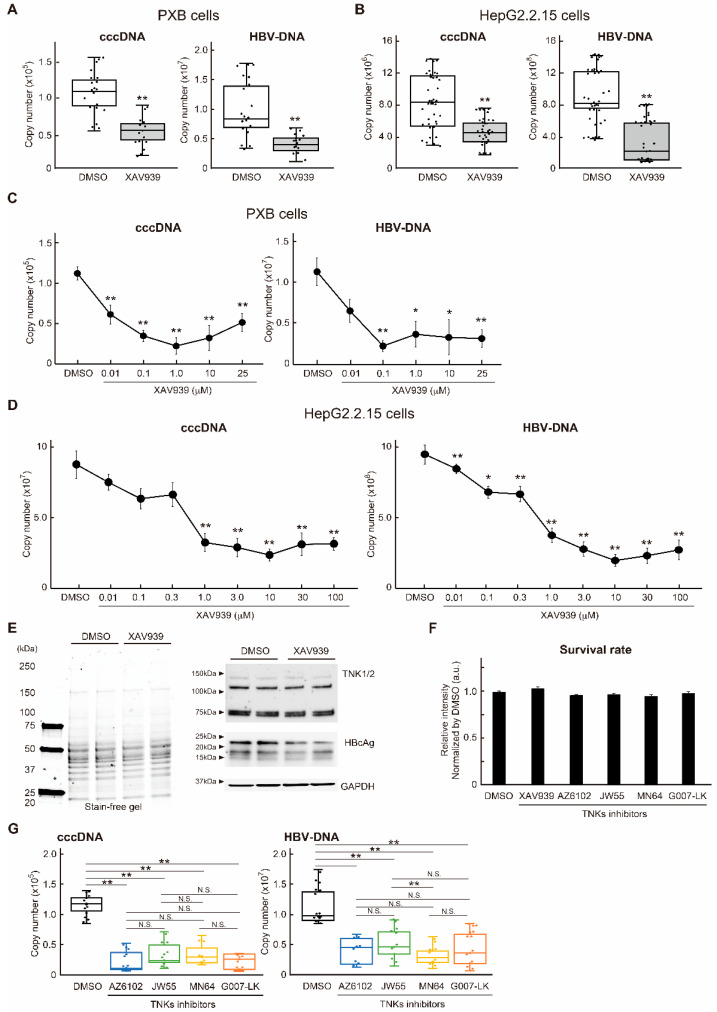
TNKS inhibitors decreased cccDNA and HBV-DNA levels. (**A**) The copy numbers of cccDNA and HBV-DNA were measured by qPCR in PXB cells. The box plot represents the median, and the box spans the interquartile range (IQR), from the 25th percentile (Q1) to the 75th percentile (Q3). Whiskers extend to 1.5 × IQR from the quartiles. To visualize the distribution of individual data points, a jitter was applied to avoid overlapping. All subsequent box-and-whisker plots follow the same convention. The concentration of XAV939 treatment was 100 nM. **, *p* < 0.01 vs. DMSO group. Number of independent cell culture batches (*N*) = 5; number of culture wells (*n*) = 16–24. (**B**) Graph shows the box-and-whisker plots of cccDNA and HBV-DNA copy numbers in HepG2.2.15 treated with DMSO or 10 μM XAV939. **, *p* < 0.01 vs. DMSO group. *N* = 8; *n* = 41–47. (**C**) Dose-dependent effects of XAV939 on cccDNA and HBV-DNA levels in PXB cells. The Y-axis indicates the raw copy number. Bars represent the mean ± S.E. *, *p* < 0.05; **, *p* < 0.01 vs. DMSO group. *N* = 3–8; *n* = 5–24. (**D**) Graph shows the dose-dependent changes in cccDNA and HBV-DNA copy numbers under DMSO or XAV939 treatment in HepG2.2.15 cells. Bars represent the mean ± S.E. *, *p* < 0.05; **, *p* < 0.01 vs. DMSO group. *N* = 5–8; *n* = 6–47. (**E**) Protein levels of TNKS, HBsAg, and GAPDH in PXB cells were determined by Western blotting. Representative blot images are shown: stain-free gel images (**left**) and corresponding immunoblot images (**right**). Each experiment was independently performed at least three times using different cell lysates. *N* = 2; *n* = 6 for each treatment group (DMSO and XAV939). (**F**) The graph shows the relative intensity (a.u.) of cell viability in each inhibitor treatment group, normalized to the DMSO-treated group. Cell viability (%) was measured by MTT assay and normalized to the mean of the DMSO group. Bars represent the mean ± S.E. No statistically significant differences were observed. *N* = 3; *n* = 8–10. (**G**) The copy numbers of cccDNA and HBV-DNA were measured by qPCR in PXB cells treated with various tankyrase inhibitors (10 nM AZ6102, 10 μM JW55, 80 nM MN64, and 50 nM G007-LK). The Y-axis indicates the raw copy number. Graph shows the box-and-whisker plots of cccDNA and HBV-DNA copy numbers in PXB cells. Notably, box-and-whisker plots are shown to illustrate the distribution of values, as sufficient variability and sample size allowed for meaningful visualization. **, *p* < 0.01 vs. DMSO or among groups as indicated. *N* = 4; *n* = 10–14. N.S.; not significant.

**Figure 4:**
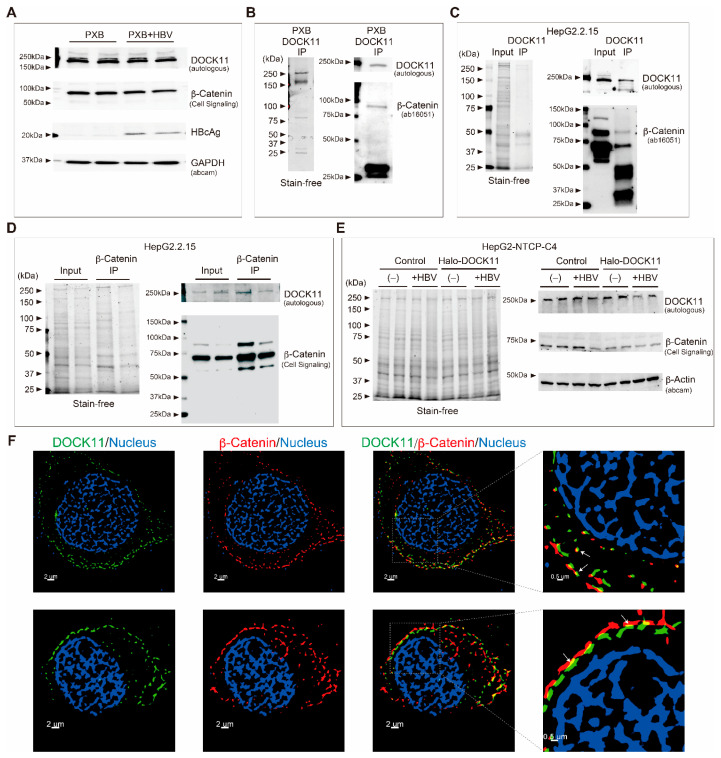
Interaction of β-catenin and DOCK11 proteins. (**A**) Protein levels of DOCK11, β-catenin, HBsAg, and GAPDH in PXB cells were determined using Western blotting. The whole blotting image obtained using stain-free gel is shown in Supplementary Figure S1. Representative blotting images for PXB control or PXB with HBV-infected groups are shown. The arrowheads indicate the size of bands (kDa). (**B**) Immunoprecipitation (IP) assays using DOCK11 in HBV-infected PXB cells. Whole gel image is shown (**left**). Western blotting using anti-DOCK11 or β-catenin antibody detected 250 kDa or 80 kDa, respectively (**right**). (**C**) IP assay using DOCK11 antibody in HepG2.2.15 cells. The whole stain-free gel image (**left**). Left lane: input; right lane: IP for DOCK11. The lysate of IP by using DOCK11 antibody was then examined for co-expression by Western blotting with β- Catenin antibodies (**right**). (**D**) IP using β-catenin antibody in HepG2.2.15 cells. Whole stain-free gel image is shown (**left**). Left lane: two inputs; right lane: two IP assays of β-catenin. DOCK11 was detected in the lysate of IP for β-catenin (**right**). (**E**) Immunoblotting of DOCK11, β-catenin, and β-actin in the cell lysate obtained from HepG2-NTCP-C4, HepG2-NTCP-C4-Halo-DOCK11 cells with or without HBV infection. Whole stain-free gel image is shown (**left**). Two representative lysates from each condition. Control; lysates from HepG2-NTCP-C4, Halo-DOCK11; lysates from HepG2-NTCP-C4-Halo-DOCK11 cells. (−); non-HBV-infected, +HBV; HBV-infected. Immunoblotting images using anti-DOCK11, β-catenin or β-Actin antibodies are shown (**right**). (**F**) Representative immunofluorescence images of HepG2.2.15 cells co-stained with DOCK11 and β-catenin antibodies. DOCK11 is shown in green, β-catenin in red, and nuclei are counterstained with DAPI (blue). Merged images demonstrate the subcellular localization and partial co-localization of DOCK11 and β-catenin. Magnified views are shown to highlight regions of co-localization. Scale bars are indicated in each image. White arrows indicate representative sites of co-localization.

**Figure 5:**
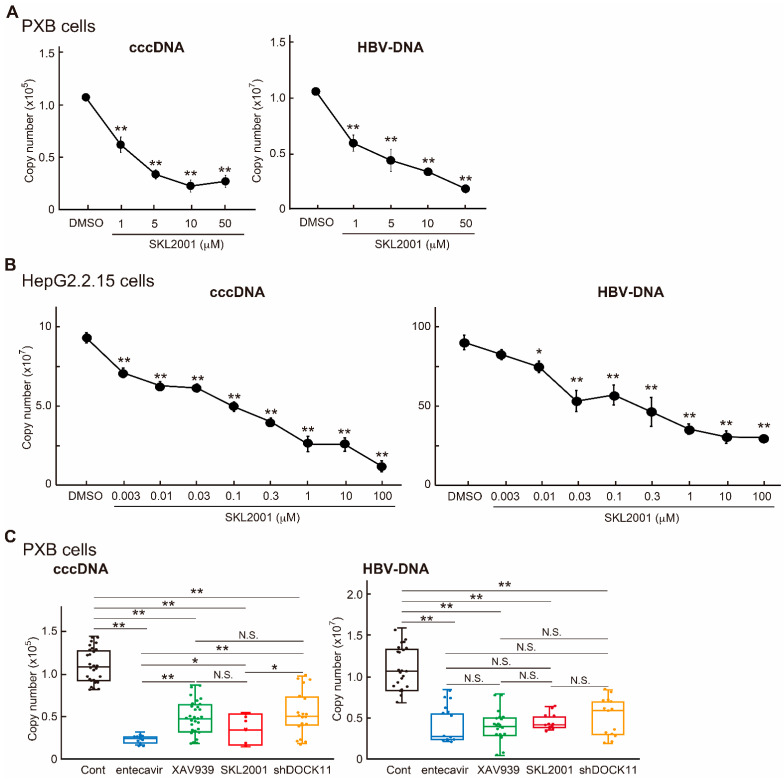
Effects of SKL2001 for eliminating cccDNA and HBV-DNA. (**A**) The effect of the Wnt/β-catenin agonist SKL2001 on cccDNA and HBV-DNA was measured by qPCR in PXB cells. The Y-axis indicates the raw copy number. Bars represent the mean ± standard error (S.E.). **, *p* < 0.01 vs. DMSO group. *N* = 3, *n* = 7–18. (**B**) Dose-dependent effects of SKL2001 on cccDNA and HBV-DNA copy numbers were assessed in HepG2.2.15 cells. The graph shows the mean ± S.E. of raw copy numbers compared to those in the 0.05% DMSO group. *, *p* < 0.05; **, *p* < 0.01 vs. DMSO group. *N* = 3, *n* = 5–8. (**C**) Graph shows the box-and-whisker plots of cccDNA and HBV-DNA copy numbers in each condition. The respective controls were purified water (for entecavir), 0.05% DMSO (for SKL2001 and XAV939), and MOCK (for shDOCK11). Comparisons were performed both against each control and among treatment groups. *, *p* < 0.05; **, *p* < 0.01; N.S., not significant. *N* = 3–4, *n* = 8–21. The final concentrations of entecavir or XAV939 were 10 nM or 100 nM, respectively.

**Figure 7:**
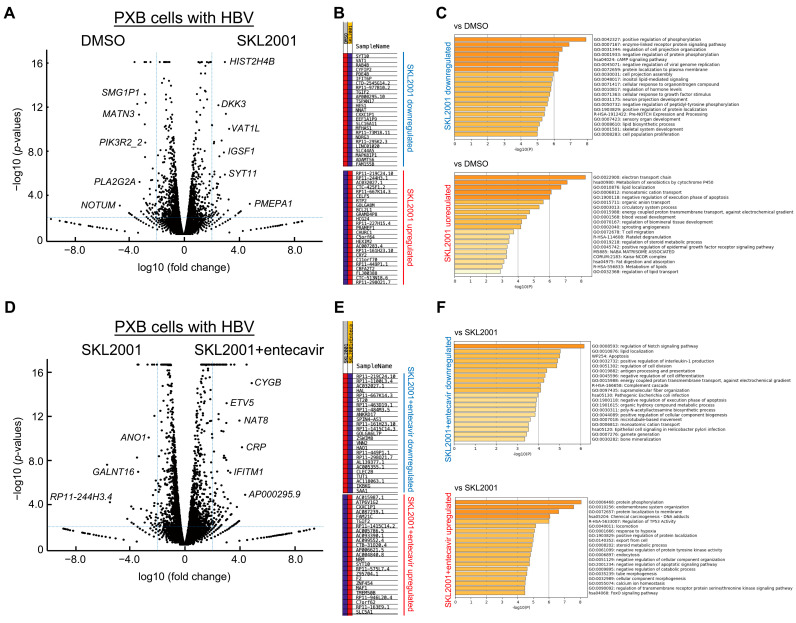
RNA-seq analysis in three different culture conditions. (**A**) RNA-seq data were analyzed by CLC Genomics Workbench, and the DEGs between DMSO and 10 μM SKL2001 treatment groups were visualized using a volcano map. The definition of DEGs (blue dotted lines) was log_2_ FC > 1.0 and −log_10_(*p*-value) > 2.0. We used two different RNA-seq datasets obtained from different PXB cells to detect DEGs. (**B**) Heatmap plots of the top 50 upregulated or downregulated DEGs obtained using gene set enrichment analysis. Each heatmap shows only the top 20 extracted genes. The colors are row-normalized rank-ordered gene scores such that the maximum value for each gene is plotted in red and the minimum value is plotted in blue. “SKL2001 downregulated” is equivalent to “DMSO upregulated,” indicating these genes might be related to the maintenance of HBV in PXB cells. (**C**) Gene Ontology (GO) analysis using Metascape showed significantly downregulated gene functions (upper graph) and upregulated gene functions (lower graph) following SKL2001 treatment. Bars indicate −log_10_(*p*-value). (**D**) Volcano map between 10 μM SKL2001 and 10 μM SKL2001 with 10 nM entecavir treatment groups. (**E**) Heatmap plots of the top 20 upregulated or downregulated DEGs obtained using GSEA enrichment analysis. “SKL2001 + Entecavir downregulated” means “SKL2001 treatment alone upregulated.” (**F**) The effect of co-treatment with SKL2001 and entecavir on signaling pathways analyzed using Metascape. Bars indicate −log_10_(*p*-value).

**Supplementary Figure S3:**
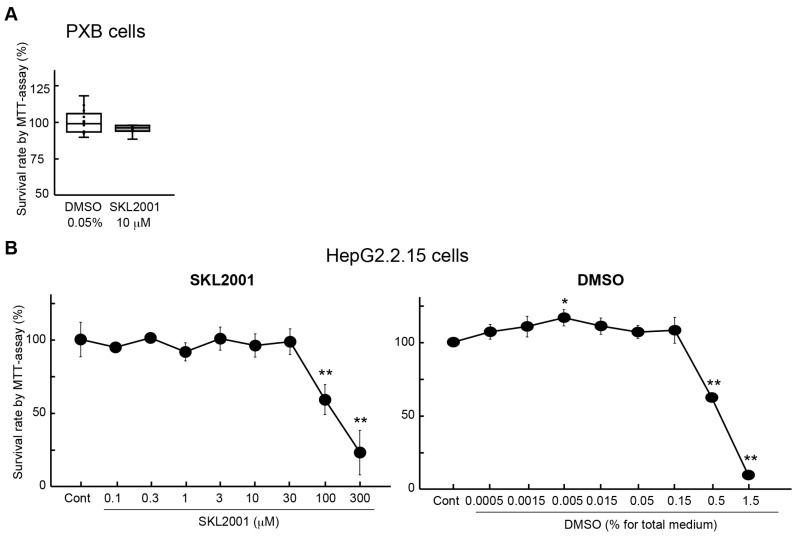
Changes in cellular cytotoxicity of dose-dependent SKL2001 treatment in PBX cells and HepG2.2.15 cells. (**A**) 10 μM SKL2001 treatment did not induce significant cell proliferation change in PXB cells. (**B**) Survival rate calculated by MTT-assay (%) in the dose-dependent manner of SKL2001 or DMSO treatment to HepG2.2.15 cells. Cont: control culture without any drugs. Bars indicate the mean ± standard error (S.E.). * or **; *p* < 0.05 or 0.01 vs. control group in each condition. Number of cell culture batch (N) = 3, number of culture wells (n) = 5−7. DMSO group: % for total medium. For example, 0.05 indicates that 0.05% of DMSO was applied to the culture medium, and the concentration of DMSO was equal to 10 μM SKL2001 treatment.

**Supplementary Figure S6:**
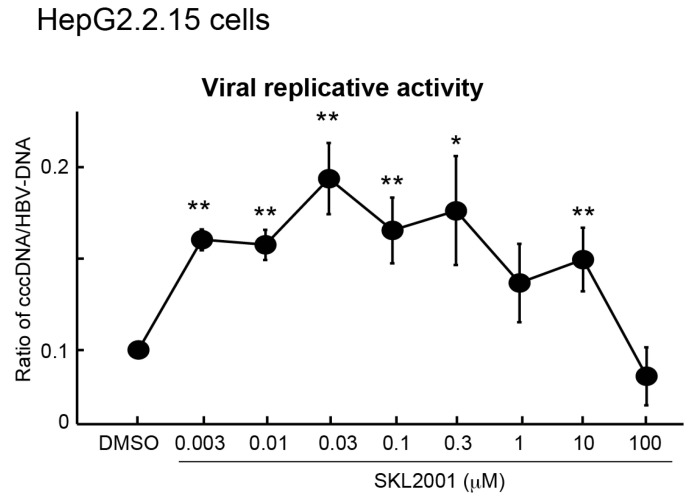
Viral replicative activity in HepG2.2.15 with dose-dependent SKL2001 treatment. The raw HBV copy numbers for cccDNA and HBV-DNA were measured; then, ratio of (HBV copy number of cccDNA)/(HBV copy number of DNA) was calculated. Bars indicate the mean ± standard error (S.E.). * or **, *p* < 0.05 or 0.01 vs. DMSO control culture. Number of cell culture batch N = 3, number of culture wells n = 5−8.
