# Supplementary material for: Wnt/β-Catenin Signaling Regulates Hepatitis B Virus cccDNA Levels
Source: Int J Mol Sci. 2025 Jul 19;26(14):6942. doi: 10.3390/ijms26146942 (PMC12295781; doi:10.3390/ijms26146942)
Supplement: Supplementary file 1 [file ijms-26-06942-s001.zip › Supplementary_Figures-mod.pdf]

6Supplementary Figures

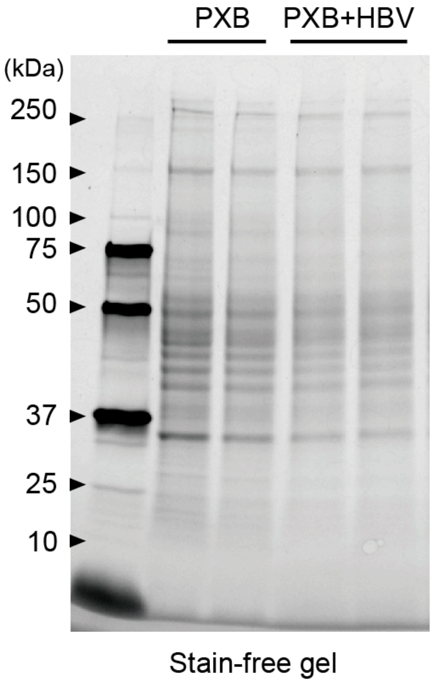

Supplementary Figure S1. Whole-extracted protein Stain-free gel image from cell lysate of PXB and HBV-infected HBV cells.

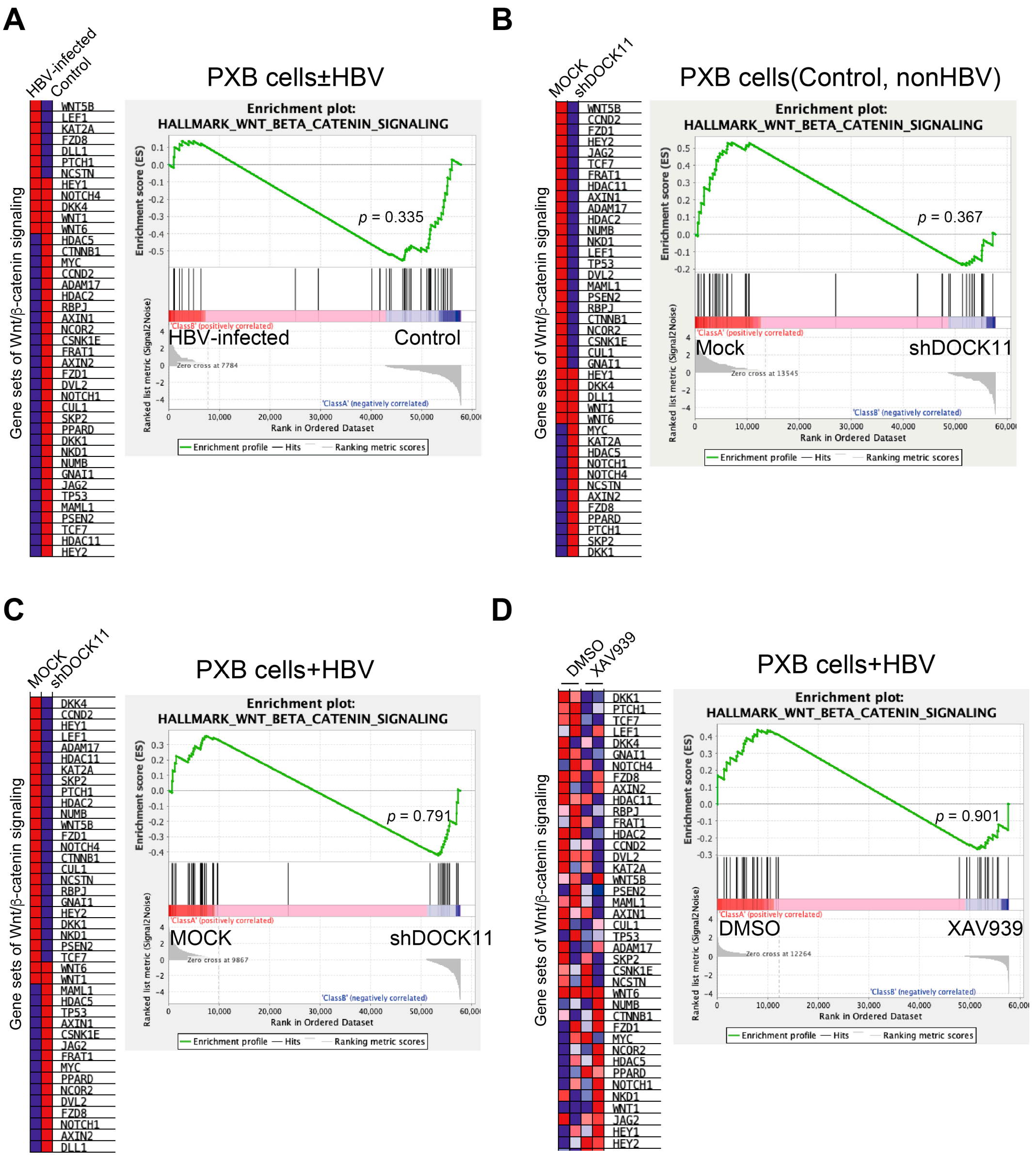

Supplementary Figure S2. Genset enrichment analysis for Wnt/β-catenin signaling in each culture condition. There were no significant differences of any comparisons. **A:** The comparison between control PXB cell and HBV-infected PXB cells. Left heatmap showed which genes enhanced in each condition related to Wnt/β-catenin signaling pathway. Red to blue scale color indicates $\log_2$ FC. Right figure showed enrichment plot by GSEA analysis. $p = 0.335$. **B:** The comparison between PXB cells transfected Mock or shRNA for DOCK11. $p = 0.367$. **C:** The comparison between HBV-infected PXB cells transfected Mock or shRNA for DOCK11. $p = 0.791$. **D:** The comparison between HBV-infected PXB cells treated by DMSO or XAV939. $p = 0.901$.

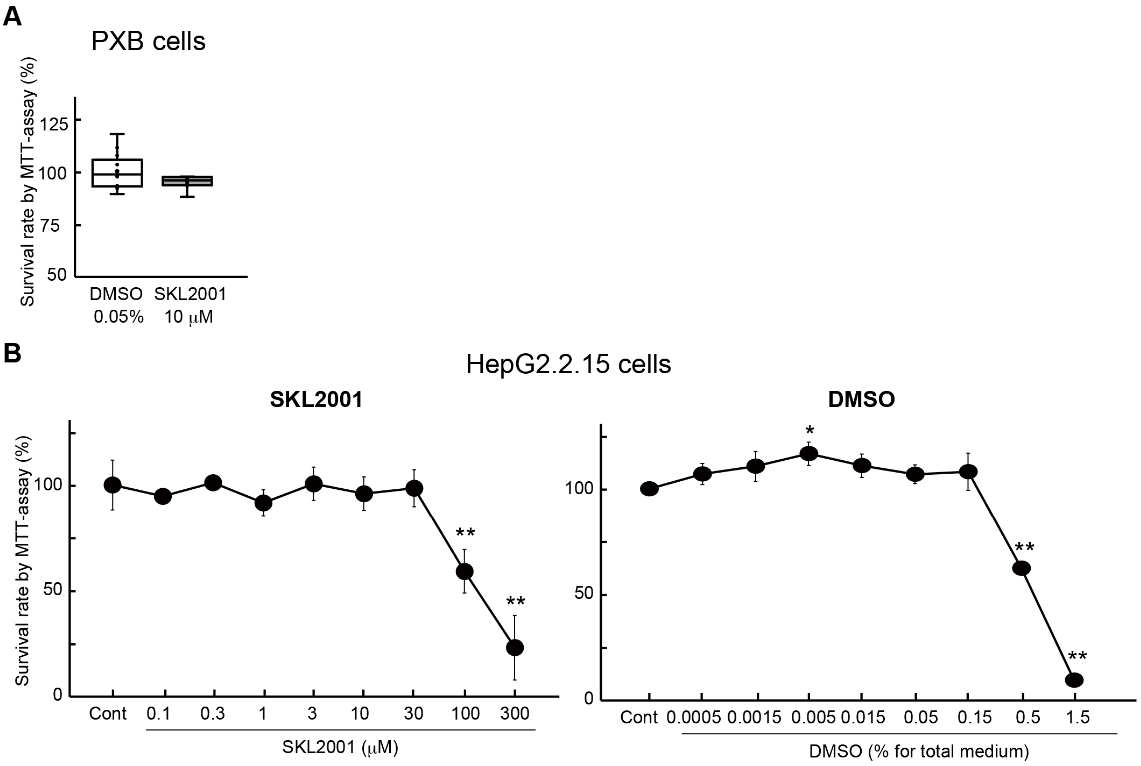

Supplementary Figure S3. Changes in cellular cytotoxicity of dose-dependent SKL2001 treatment in PBX cells and HepG2.2.15 cells. (**A**) 10 μM SKL2001 treatment did not induce significant cell proliferation change in PXB cells. (**B**) Survival rate calculated by MTT-assay (%) in the dose-dependent manner of SKL2001 or DMSO treatment to HepG2.2.15 cells. Cont: control culture without any drugs. Bars indicate the mean ± standard error (S.E.). * or **; $p < 0.05$ or 0.01 vs. control group in each condition. Number of cell culture batch (N) = 3, number of culture wells (n) = 5−7. DMSO group: % for total medium. For example, 0.05 indicates that 0.05% of DMSO was applied to the culture medium, and the concentration of DMSO was equal to 10 μM SKL2001 treatment.

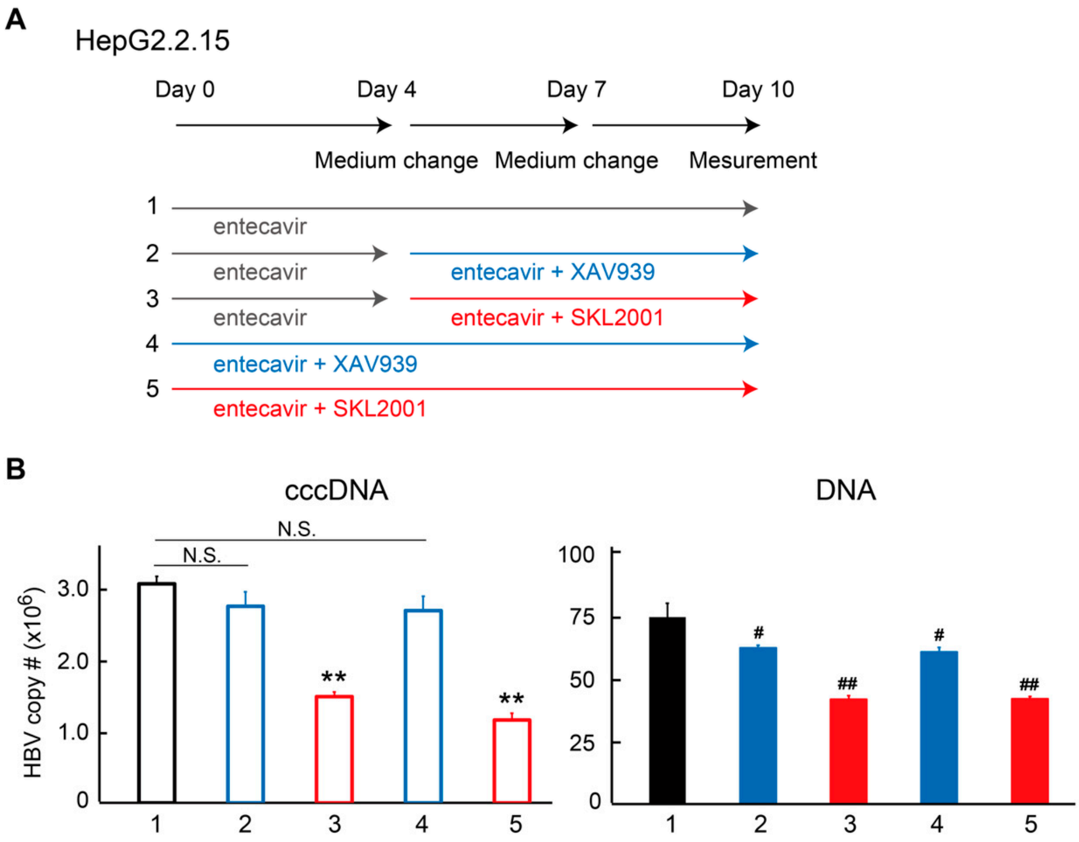

Supplementary Figure S4. Combination drug effects of Entecavir in HepG2.2.15 cells. **A:** The loading status of various inhibitors to cell culture. (1) Entecavir treatment throughout culture, (2) or (3) Entecavir treatment until Day4, then Entecavir with XAV939 (blue) or SKL2001 treatment (red), (4) or (5) Entecavir with XAV939 (blue) or SKL2001 (red) treatment throughout culture. **B:** The bar graphs show effect of combination treatment in different culture condition. Y axis indicated raw HBV copy number ($\times 10^6$). The bordered graph shows cccDNA, and the filled graph shows HBV-DNA. **, $p < 0.01$ vs culture condition #1 for cccDNA. $^{\#}$ or $^{\#\#}$, $p < 0.05$ or 0.01 vs culture condition #1 for HBV-DNA. Number of culture batch N=3, culture well n=8−14.

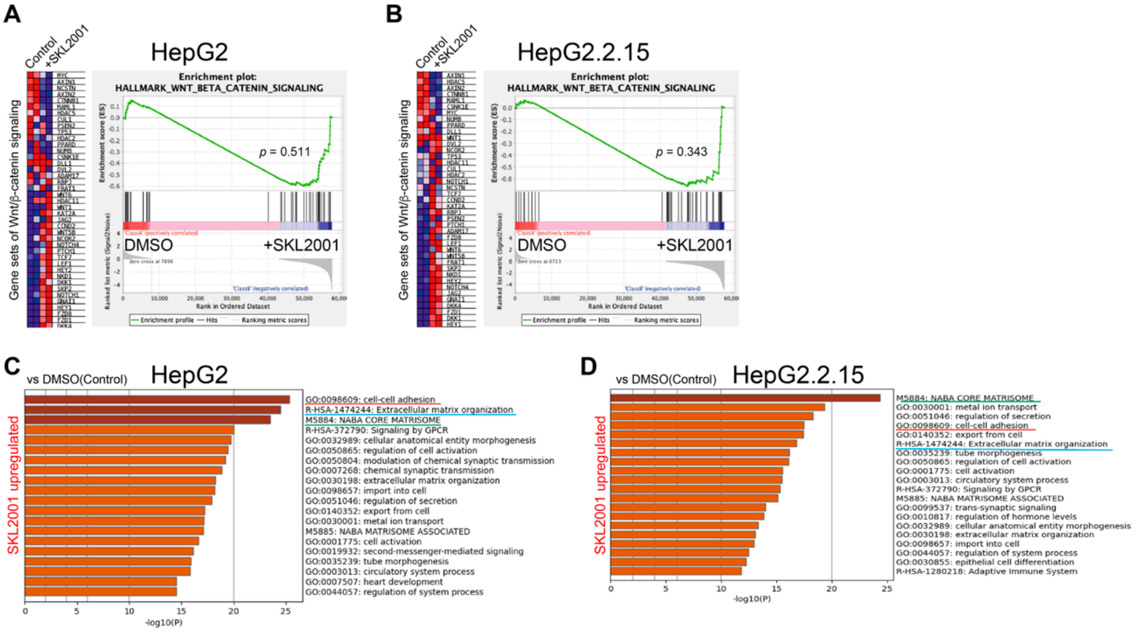

Supplementary Figure S5. Gene set analysis of RNA-seq results in non-HBV HepG2 and HepG2.2.15 cell culture with SKL2001 treatment. **A or B:** GSEA analysis for Wnt/β-catenin signaling in HepG2 or HepG2.2.15 cells. Left heatmap showed which genes enhanced in each condition related to Wnt/β-catenin signaling pathway. Red to blue scale color indicates $\log_2$ FC. Right figure showed enrichment plot by GSEA analysis. p = 0.511 or 0.343. **C or D:** Gene Ontology (GO) analysis by Metascape showed significantly upregulated gene functions for SKL2001 treatment in each cell line. The bars indicate -$\log_{10}$(p-value). The TPM value of a gene in SKL2001 treatment group was normalized by that in DMSO (Control) group. The value > 1.5 set as threshold.

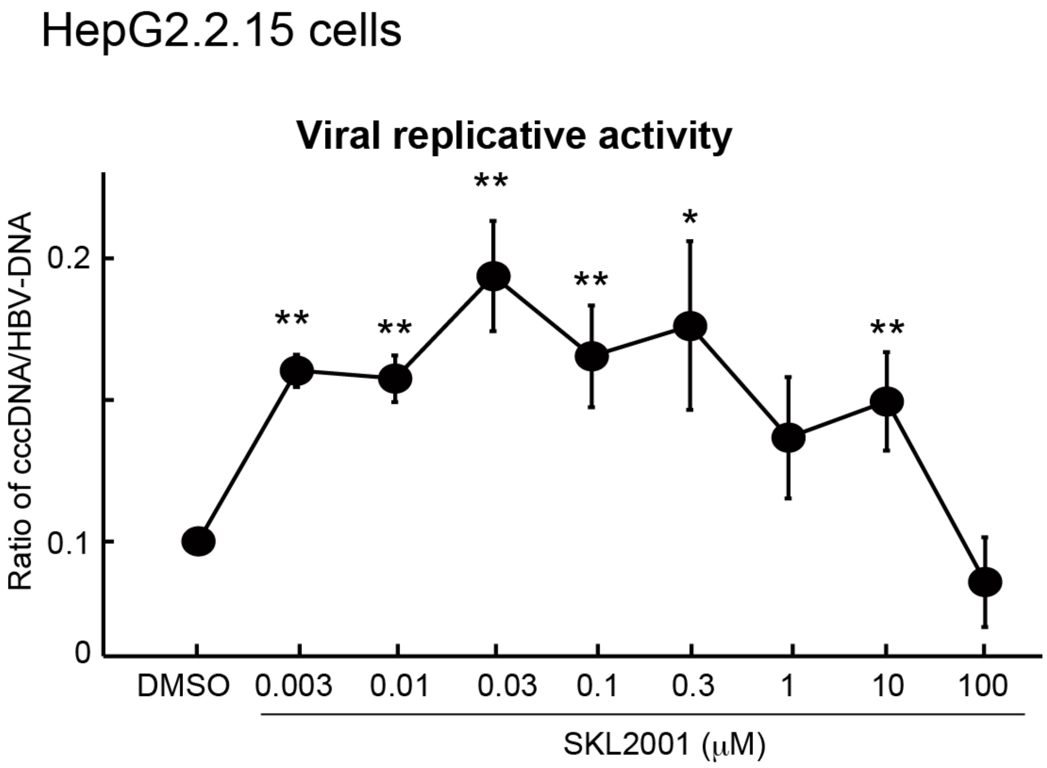

Supplementary Figure S6. Viral replicative activity in HepG2.2.15 with dose-dependent SKL2001 treatment. The raw HBV copy numbers for cccDNA and HBV-DNA were measured; then, ratio of (HBV copy number of cccDNA)/(HBV copy number of DNA) was calculated. Bars indicate the mean ± standard error (S.E.). * or **, $p < 0.05$ or 0.01 vs. DMSO control culture. Number of cell culture batch N = 3, number of culture wells n = 5−8.
